# Urine-derived stem cells efficiently assemble into micro-bone organoids supported by decellularized bone matrix microparticles for rapidly repairing bone defects through direct filling and paracrine functions

**DOI:** 10.1016/j.mtbio.2025.102533

**Published:** 2025-11-07

**Authors:** Yiting Chen, Liang Zhang, Zeyu Li, Xinrun Wang, Jie Liu, Xianwen Wang, Jiyun Hu, Guotao Wang, Qihang Huang, Yuhao Yuan

**Affiliations:** a Department of Orthopedics, Xiangya Hospital, Central South University, Changsha, Hunan, 410008, China; b Department of Critical Care Medicine, Xiangya Hospital, Central South University, Changsha, Hunan, 410008, China; c National Clinical Research Center for Geriatric Disorders, Xiangya Hospital, Central South University, Changsha, Hunan, 410008, China; d Department of Orthopedic Trauma, Chenzhou No. 1 People's Hospital, Chenzhou, Hunan, 423000, China

**Keywords:** Urine-derived stem cells, Decellularized bone matrix, Bone organoid, Paracrine functions, Bone defect

## Abstract

The repair of large bone defects remains a significant challenge in orthopedic clinical practice. This study aims to rapidly cultivate a novel type of bone organoids (BOs), namely uBOs (USCs@DBM-MPs derived BOs), by utilizing self-developed highly biomimetic decellularized bone matrix microparticles (DBM-MPs) as the supporting carrier in combination with non-invasively obtained urine-derived stem cells (USCs), and to explore its therapeutic efficacy and biological mechanism. In our vitro experiments confirmed that DBM-MPs exhibit excellent biocompatibility and osteoinductivity, and urine-derived stem cells (USCs) have comparable osteogenic potential to bone marrow stem cells (BMSCs). Furthermore, USCs were loaded onto DBM-MPs for osteogenic directional induction, and a novel bone organoid—uBOs, was successfully generated within 14 days. Meanwhile, compared with bBOs (BMSCs@DBM-MPs derived BOs), uBOs exhibit comparable levels of biological activity, proliferation characteristics, and osteogenic potential. Moreover, uBOs offer the advantages of a broader availability and a non-invasive acquisition process. What is particularly noteworthy is that these uBOs exhibit paracrine functions capable of promoting both angiogenesis and osteogenesis. In-vivo rat femoral condyle defect model, minimally invasive injection of uBOs into the bone defect area achieved rapid bone regeneration within only 6 weeks, perfectly repairing the defect area. The uBOs developed in this study not only as a bone substitute unit for direct filling and repair of bone defects, but also continuously induce angiogenesis and bone fusion at the defect site through their paracrine mechanism, offering a brand-new and efficient tissue engineering strategy for bone defect treatment.

## Highlights

• USCs supported by DBM-MPs, can efficiently generate novel micro-bone organoids (uBOs).
• uBOs can function as off-the-shelf substitutes to completely fill bone defects and achieve rapid bone repair.
• uBOs demonstrate paracrine functions capable of stimulating angiogenesis and osteogenesis.

## Graphical abstract

Decellularized bone matrix (DBM) was initially prepared using an independent decellularization system. Subsequently, a novel bone organoid carrier—decellularized bone matrix microparticles (DBM-MPs), characterized by high biomimicry, easy accessibility, and superior osteoinductivity, was constructed via freeze-milling and filtration. Urine-derived stem cells (USCs) with non-invasive, widely available, and sustainable were utilized as the seed cells for bone organoid construction. Furthermore, USCs were loaded onto DBM-MPs for osteogenic directional induction, and a novel bone organoid—uBOs, was successfully generated within 14 days. These uBOs demonstrate paracrine functions capable of stimulating angiogenesis and osteogenesis, and can function as off-the-shelf substitutes to completely fill bone defects and achieve rapid bone repair.

**Figure:**
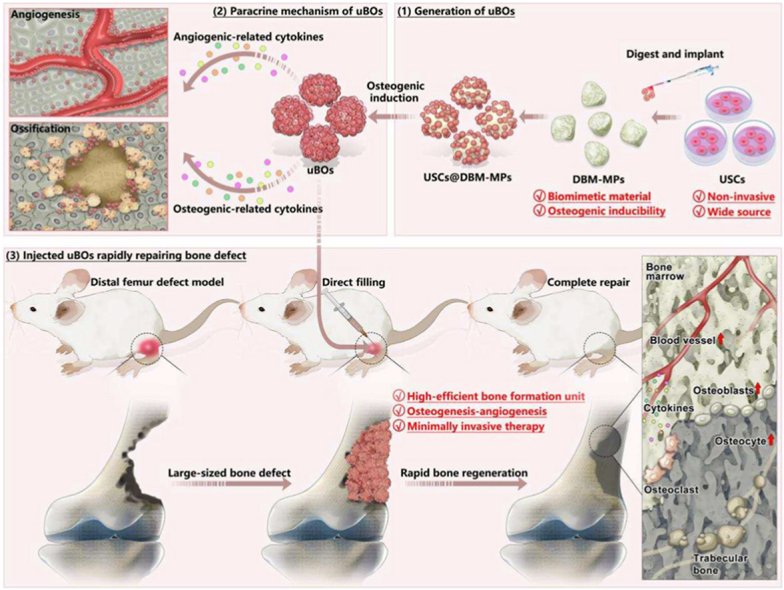

## 1 Introduction

The repair of bone defects caused by trauma, tumors, infections, etc. has always been a major challenge in clinical orthopedics [1]. Especially when the degree of bone defect exceeds the critical size threshold, that is, the length is > 2 cm or the bone circumference loss exceeds 50 % [2]. In such cases, the bone tissue lacks sufficient self-regeneration ability, without external intervention, it will lead to non-union or poor healing of the bone, and thus bone grafting surgery is often required [3]. Among available options, autologous bone grafting is widely regarded as the gold standard for treating bone defects due to its excellent osteoconductive and osteogenic properties, as well as the absence of disease transmission risks. Nevertheless, this approach inevitably introduces complications at the donor site, including trauma, pain, and hematoma formation. Furthermore, the source of autologous bone is limited, it cannot be arbitrarily reshaped, and postoperative bone resorption rates are unpredictable, thereby restricting its application [4,5]. Additionally, while allogeneic bone grafting exhibits some degree of osteoconductivity, it poses challenges such as immune rejection, low osteoinductive activity, and potential cross-disease transmission [6]. Consequently, bone tissue engineering (BTE), which integrates scaffolds, cells, and bioactive factors as its three core elements, has emerged as a promising strategy for developing ideal bone substitutes [7,8].

Recently, the advent of organoid technology has provided a brand-new strategy for BTE [9]. Different from other BTE products, organoids are derived from adult stem cells, pluripotent stem cells, induced pluripotent stem cells, etc, with the support of conditions, they differentiate directionally into three-dimensional cell clusters [10]. The structural and functional characteristics of organoids exhibit a remarkable degree of similarity to those of the target organs. Therefore, they can act as bone substitute units in bone defect repair [11,12]. Specifically, bone organoids (BOs) can directly fill the defect area while also actively chemoattracting surrounding bone tissues to migrate and fuse, thereby promoting rapid bone regeneration [13]. Furthermore, from the perspective of biotransformation, the in-vitro cultivation of BOs not only more accurately recapitulates the complex microenvironment of bone tissue but also enables large-scale production. When applied to the treatment of bone defects in-vivo, it can be reconstructed in a minimally invasive and injectable manner [14]. Consequently, the research, development, and construction of novel BOs hold exceptionally high clinical significance for the future treatment of extensive bone defects.

The construction of BOs still relies on seed cells and three-dimensional directional culture systems [15,16]. Therefore, in this study, considering from the perspectives of non-invasive, wide source, and sustainability, we propose to select urine-derived stem cells (USCs) as the seed cells for constructing the novel BOs. USCs are a type of adult stem cells isolated from urine with the biological characteristics of mesenchymal stem cells [17]. In recent years, USCs have attracted increasing attention from tissue engineering researchers. In previous related literature reports, it has been confirmed that USCs can be differentiated into various types of cells such as bone cells and chondrocytes after induction [[18], [19], [20]]. Interestingly, some scholars have combined USCs with GelMA hydrogels, 3D scaffolds, electrospun scaffolds, etc. for bone defect repair, and have also achieved satisfactory results [[20], [21], [22]]. In these studies, compared with the simple scaffold group, the experimental group combined with USCs has more efficient bone repair efficacy. However, it is still unclear whether USCs can be used to cultivate BOs and whether they can further enhance their bone induction performance. More importantly, compared with other stem cells, the isolation and acquisition process of USCs is completely non-invasive, simple, fast, and is obtained from the normal metabolic waste urine of the body [23]. Thus, the cultivation of novel BOs based on USCs may achieve turning waste into treasure in the BTE strategy, which has no relevant literature reports before and has considerable research value.

Of course, the cultivation of BOs heavily relies on the support of three-dimensional carriers [24,25]. In prior studies, researchers frequently utilized biomaterials such as Matrigel, GelMA microspheres, silk fibroin, and highly polymerized compounds for bone organoid development [14,26,27]. These materials have been shown to effectively facilitate the adhesion and proliferation of stem cells. Within an in-vitro directed induction system, these cell-carrier complexes can autonomously differentiate and proliferate, gradually forming bone microtissues. Nevertheless, from a histological perspective, these carriers fail to adequately mimic the complex microenvironment of bone tissue [28,29]. Moreover, their compositions differ markedly from those of the natural bone matrix, which inevitably compromises the biomimetic properties of BOs to some extent [30,31]. Consequently, it is imperative to investigate three-dimensional carriers with higher bionic characteristics and superior biological activity. The emergence of decellularization technology offers a novel approach to this challenge [32]. This biotechnology removes cells and immunogenic nuclear components from tissues or organs while preserving the natural extracellular matrix constituents [33,34]. Therefore, through the decellularization process, it may be feasible to develop highly biomimetic carriers that exhibit low immunogenicity, high biocompatibility, favorable biodegradability, and superior biological activity [35], namely decellularized bone matrix (DBM). Theoretically, DBM mimics the natural microenvironment, which contains microstructure and active substances similar to the primary target tissue. This creates an optimal environment for stem cell adhesion, proliferation, and functional differentiation. Furthermore, in our study, the blocky DBM was innovatively processed into particulate form by freeze-grinding and filtration, namely the microparticles of DBM (DBM-MPs). These DBM-MPs not only retain the bionic nature and biological activity of the blocky DBM, but also facilitate the exposure of matrix proteins, thereby efficiently stimulating stem cells to differentiate into bone cells. Meanwhile, these micron-sized particles can be delivered to the defect site through minimally invasive injection for complete filling. Therefore, this study hypothesizes that using DBM-MPs as a novel cultivation carrier for BOs, loading the seed cells USCs, and conducting in-vitro osteogenic directional induction, may provide a highly bionic, widely sourced, easily accessible, and readily cultivable novel bone organoid for the treatment of bone defects.

**Fig. 1:**
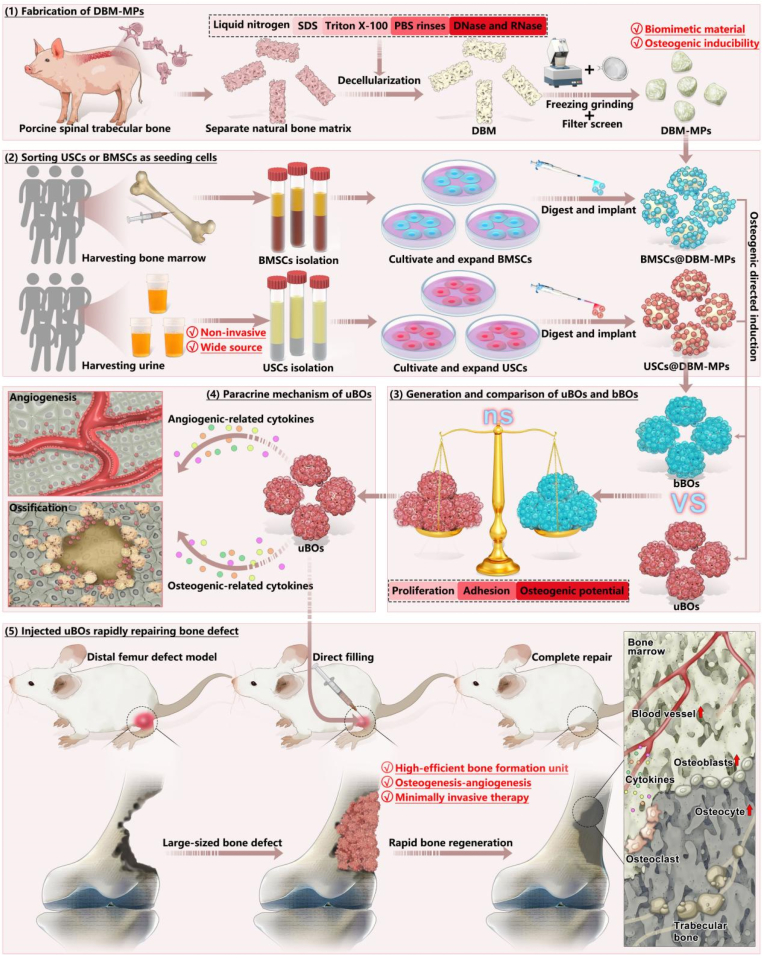
By utilizing a self-constructed decellularization system to fabricate DBM-MPs with high biomimetic properties and excellent osteogenic performance as the supporting carrier, combined with non-invasively obtained USCs for rapid cultivation, a novel type of bone organoid (uBOs) was developed. Furthermore, evaluating the morphological characteristics, genetic phenotypes, biological mechanisms and *in vivo* performance of uBOs.

Overall, in this study (Fig. 1), we aimed to fabricate highly biomimetic DBM-MPs using a self-constructed decellularization system. Subsequently, bone marrow mesenchymal stem cells (BMSCs) and USCs were isolated and expanded. These two cell types were separately loaded onto DBM-MPs (referred to as BMSCs@DBM-MPs and USCs@DBM-MPs), followed by in-vitro osteogenic induction culture. This process yielded BOs constructed from BMSCs and DBM-MPs (denoted as bBOs) and those constructed from USCs and DBM-MPs (denoted as uBOs). Using bBOs as the positive control, we investigated the osteogenic induction performance and underlying biological mechanisms of uBOs. Furthermore, the in-vivo repair capability and regenerative efficacy of the novel bone organoid uBOs were validated using the rat femoral condyle bone defect model.

## 2 Results

### 2.1 Preparation and characterization of DBM

**Fig. 2:**
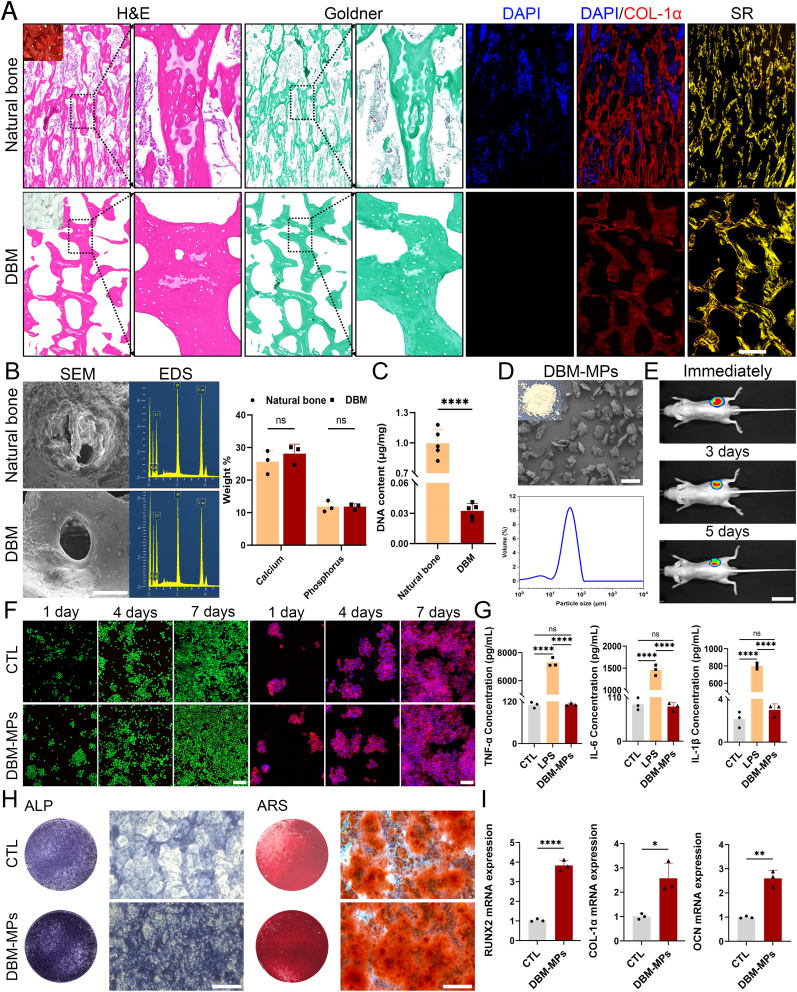
**Preparation and Functional Characterization of DBM-MPs.** (A) H&E, Goldner, DAPI/*COL-1α*, and SR staining were performed to assess alterations in the bone matrix before and after decellularization. Scale bar = 100 μm. (B) Observe the microstructure and calcium/phosphorus content variations before and after decellularization using SEM and EDS. Scale bar = 100 μm. (C) The DNA content in bone tissue before and after decellularization. (D) Microstructure and particle size detection of DBM-MPs. Scale bar = 50 μm. (E) Fluorescence imaging results in nude mice following subcutaneous injection of DBM-MPs at immediate, 3rd day, and 5th day time points. Scale bar = 1 cm. (F) Live/dead staining and DAPI/phalloidin fluorescence staining of RAW264.7 cells co-cultured with DBM-MPs for 1, 4, and 7 days. Scale bar = 25 μm or 50 μm. (G) On the 4th day of co-culture of RAW264.7 cells and DBM-MPs, ELISA is used to detect the secretion of inflammatory factors *TNF-α*, *IL-6*, and *IL-1β* in the culture system. (H) After 14 days of osteogenic induction, ALP staining is used to evaluate the osteogenic differentiation of BMSCs stimulated by DBM-MPs. After 21 days of osteogenic induction, ARS staining is used to detect calcium nodules in each group. Scale bar = 250 μm. (I) The osteogenic (*RUNX2*, *COL-1α*, *OCN*) genes expressed in BMSCs under DBM-MPs stimulation evaluated using qRT-PCR analysis. Data are presented as mean ± standard deviation (SD) (n = 3 or 5). p-values are calculated using unpaired *t*-test. ∗p < 0.05, ∗∗p < 0.01, ∗∗∗∗p < 0.0001, and “ns” indicates no significance.

At the local slaughterhouse, cancellous bone blocks were harvested from the pig's vertebrae and subsequently subjected to decellularization treatment as described in our previous study. Typically, the natural bone prior to decellularization was rich in blood and bone marrow, whereas the DBM following decellularization exhibited a white and porous structure. Hematoxylin-Eosin (H&E) and Goldner staining revealed a high density of cellular components within the natural bone. In contrast, the DBM exhibited an absence of cellular elements both within and surrounding the bone matrix, while maintaining a continuous and intact trabecular bone matrix structure (Fig. 2A). Further, DAPI/*COL-1α* fluorescence staining and Sirius red (SR) staining reveal that the nuclear materials in DBM have been thoroughly removed, and compared with natural bone, DBM retains a substantial amount of active collagen matrix components (Fig. 2A). Scanning Electron Microscope (SEM) analysis revealed that the micropores in DBM were smoother and free of impurities compared to those in natural bone. Energy Dispersive Spectroscopy (EDS) detection further confirmed that the ratio of calcium and phosphorus substances in decellularized DBM closely resembled that of natural bone (Fig. 2B). Simultaneously, quantitative DNA analysis was performed on both DBM and natural bone. The results demonstrated that the DNA content in DBM was extremely low, at 0.0324 ± 0.00726 μg/mg (Fig. 2C), which is significantly lower than the minimum threshold of 0.05 μg/mg for inducing immune rejection reactions in previous literature [36,37]. In summary, the DBM obtained through the independently constructed decellularization system in this study has been thoroughly cleared of cellular and nuclear material components, while effectively retaining the active matrix and inorganic components such as calcium and phosphorus.

### 2.2 Fabrication and biological functions of DBM-MPs

The bulk DBM obtained was cryogenically ground into a powder and subsequently sieved through a 80 μm aperture steel wire mesh to prepare DBM-MPs. Visually, DBM-MPs appeared as a white powder. Further SEM analysis indicated that the morphology of DBM-MPs consisted of irregular granules with relatively uniform size distribution. Particle size measurements revealed that nearly all particles ranged between 10 μm and 100 μm in diameter, with approximately 95 % of the particle diameters were less than 80 μm (Fig. 2D). Meanwhile, immediate *in vivo* fluorescence imaging results of the subcutaneous simulated injection model in nude mice showed a strong clustered fluorescent signal localized at the injection site. Notably, the fluorescence remained predominantly confined to the subcutaneous tissue on day 3 and day 5 post-injection, indicating sustained local retention and supporting the minimally invasive injectability of DBM-MPs (Fig. 2E). These characteristics align well with the requirements for use as a carrier in bone organoid applications.

The biocompatibility of DBM-MPs was assessed through co-culture experiments with RAW264.7 macrophage cells. Specifically, on days 1, 4, and 7, cell viability was evaluated by live/dead staining between the DBM-MPs co-culture group and the control (CTL) group. The results demonstrated that cells in both groups exhibited continuous proliferation without significant differences. At all time points, there were no obvious dead cells stained with red fluorescence, while the majority of cells were stained green fluorescence, indicating high viability. Additionally, DAPI/phalloidin fluorescence staining revealed a healthy cytoskeletal structure and favorable growth state for RAW264.7 cells in both the DBM-MPs and CTL groups, with no signs of inflammatory polarization (Fig. 2F). On day 4, Enzyme linked immunosorbent assay (ELISA) analysis of the cell supernatant indicated that RAW264.7 cells in the lipopolysaccharides (LPS)-treated positive control group exhibited significant inflammatory activation, with elevated secretion of *TNF-α*, *IL-6*, and *IL-1β*. In contrast, the expression levels of these inflammatory cytokines in the DBM-MPs group were comparable to those in the CTL group, showing no statistically significant differences (Fig. 2G). Furthermore, the Cell Counting Kit-8 (CCK-8) results showed that the proliferation curves of BMSCs co-cultured with DBM-MPs were similar to those of CTL, with no significant differences (Fig. S1). These findings confirm that the DBM-MPs developed in this study possess excellent biocompatibility, exhibit no cytotoxicity, and do not induce inflammatory responses in RAW264.7 cells.

By establishing a co-culture system with BMSCs, the osteogenic performance of DBM-MPs was evaluated. After 14 days of osteogenic induction, alkaline phosphatase (ALP) staining was conducted, revealing that the ALP activity in the co-culture group containing DBM-MPs was significantly higher than that in the CTL group, as evidenced by a deeper blue-purple color under microscopy. Additionally, Alizarin Red S (ARS) staining was performed 21 days after osteogenic induction. The results demonstrated that while the CTL group exhibited a certain amount of red-stained calcium nodules, the DBM-MPs group showed greater deposition of red-stained calcium nodules and more extensive mutual fusion of these nodules (Fig. 2H). Furthermore, quantitative real-time PCR (qRT-PCR) analysis revealed that the expression levels of osteogenesis-related genes (*RUNX2*, *COL-1α*, *OCN*) were significantly elevated in the DBM-MPs group compared to the CTL group (Fig. 2I). Collectively, these findings indicate that BMSCs stimulated by DBM-MPs exhibit stronger osteogenic potential.

### 2.3 Compare the osteogenic potential of USCs and BMSCs, as well as their efficient loading capacity of DBM-MPs

As illustrated in Fig. 2A, waste urine and bone marrow fluid were separately collected from the human body to extract USCs and BMSCs, respectively. The osteogenic potential of these two cell types was then compared. Furthermore, after loading them onto DBM-MPs and culturing them under standard in-vitro conditions, comparisons were made regarding cell viability, proliferation, and adhesion between USCs@DBM-MPs and BMSCs@DBM-MPs. Simultaneously, under in-vitro osteogenic induction conditions, the novel uBOs and bBOs were successfully constructed. The osteogenic induction activities of the two were subsequently compared through biomolecular experiments.

**Fig. 3:**
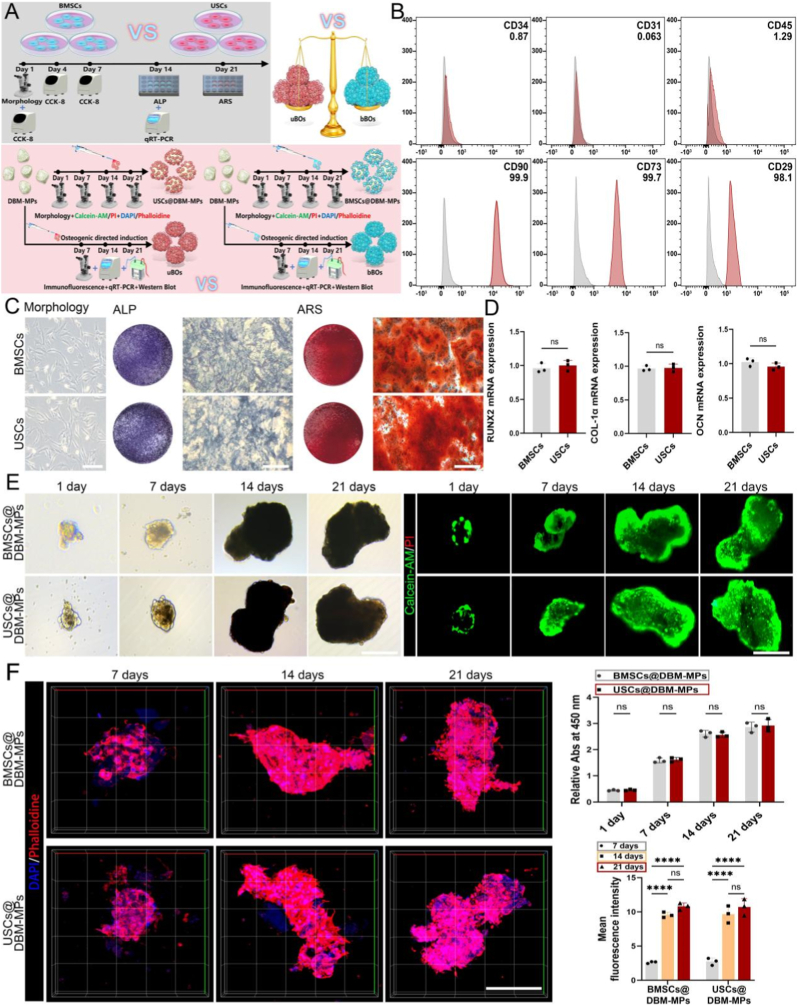
**Compare the osteogenic potential of USCs and BMSCs, as well as their efficient loading capacity of DBM-MPs.** (A) Schematic illustration depicting the functional comparison among USCs and BMSCs, USCs@DBM-MPs and BMSCs@DBM-MPs, uBOs and bBOs. (B) Flow cytometric identification of surface markers of stem cells. (C) At 14 days and 21 days of osteogenic induction, compare the osteogenic potential of USCs and BMSCs using ALP staining and ARS staining. Scale bar = 50 μm or 250 μm. (D) At 14 days post-osteogenic induction, the expression levels of osteogenic markers (*RUNX2*, *COL-1α*, *OCN*) were assessed in USCs and BMSCs by qRT-PCR. (E) The growth of USCs@DBM-MPs and BMSCs@DBM-MPs were observed using optical microscopy and live/dead cell staining. Scale bar = 50 μm. (F) Use DAPI/phalloidin three-dimensional fluorescence imaging and CCK-8 analysis to evaluate the adhesion and proliferation ability of USCs@DBM-MPs and BMSCs@DBM-MPs. Scale bar = 50 μm. Data are presented as mean ± SD (n = 3). p-values are calculated using unpaired *t*-test. ∗∗∗∗p < 0.0001, and “ns” indicates no significance.

Firstly, flow cytometry analysis of stem cell surface markers confirmed that the extracted USCs exhibited high expression of positive markers *CD90*, *CD73*, and *CD29*, while negative markers *CD34*, *CD31*, and *CD45* were not detected (Fig. 3B). Using BMSCs as a control, ALP staining was conducted after 14 days of osteogenic induction. The results demonstrated comparable blue-purple staining intensity between the USCs and BMSCs groups. Following 21 days of osteogenic induction, ARS staining revealed a similar number of calcium nodules deposited in both the USCs and BMSCs groups, with no significant difference observed (Fig. 3C). Meanwhile, qRT-PCR analysis performed 14 days post-induction showed that the expression levels of osteogenic markers (*RUNX2*, *COL-1α*, *OCN*) were closely matched between the USCs and BMSCs groups, with no statistical difference noted (Fig. 3D). Collectively, these findings suggest that the in-vitro osteogenic potential of USCs is comparable to that of BMSCs.

Additionally, USCs or BMSCs were loaded onto DBM-MPs to form USCs@DBM-MPs and BMSCs@DBM-MPs. Subsequently, during co-culture on the 1st, 7th, 14th, and 21st days, optical microscopy revealed that by the 1st day, USCs or BMSCs had already been fully loaded onto DBM-MPs. Moreover, the morphology of USCs@DBM-MPs and BMSCs@DBM-MPs gradually expanded from the 1st day to the 14th day, while their volume stabilized between the 14th and 21st days. This phenomenon may be attributed to chemotactic interactions within the cell matrices during the early culture period of USCs/BMSCs@DBM-MPs, which promoted mutual aggregation of the USCs/BMSCs@DBM-MPs. However, in the later stages, both the chemotactic effects and the loading capacity of DBM-MPs reached saturation, leading to a stable volume size. Meanwhile, the cell viability of USCs@DBM-MPs and BMSCs@DBM-MPs was assessed using live/dead cell staining. At all time points, both groups exhibited, excellent cell viability, with nearly all cells appearing as live cells stained with green fluorescence. The morphological characteristics observed were consistent with those previously identified under the optical microscope (Fig. 3E). Similarly, three-dimensional fluorescence imaging using DAPI/phalloidin demonstrated that DBM-MPs exhibited excellent adsorption properties for both USCs and BMSCs. In the early stages, USCs and BMSCs displayed a sustained proliferation state on DBM-MPs, eventually reaching saturation in the later stages. CCK-8 analysis further confirmed this trend, revealing no significant difference in proliferation capacity between the USCs@DBM-MPs and BMSCs@DBM-MPs groups (Fig. 3F).

### 2.4 In-vitro cultivation of novel BOs (uBOs) and assessment of its ossification potential

**Fig. 4:**
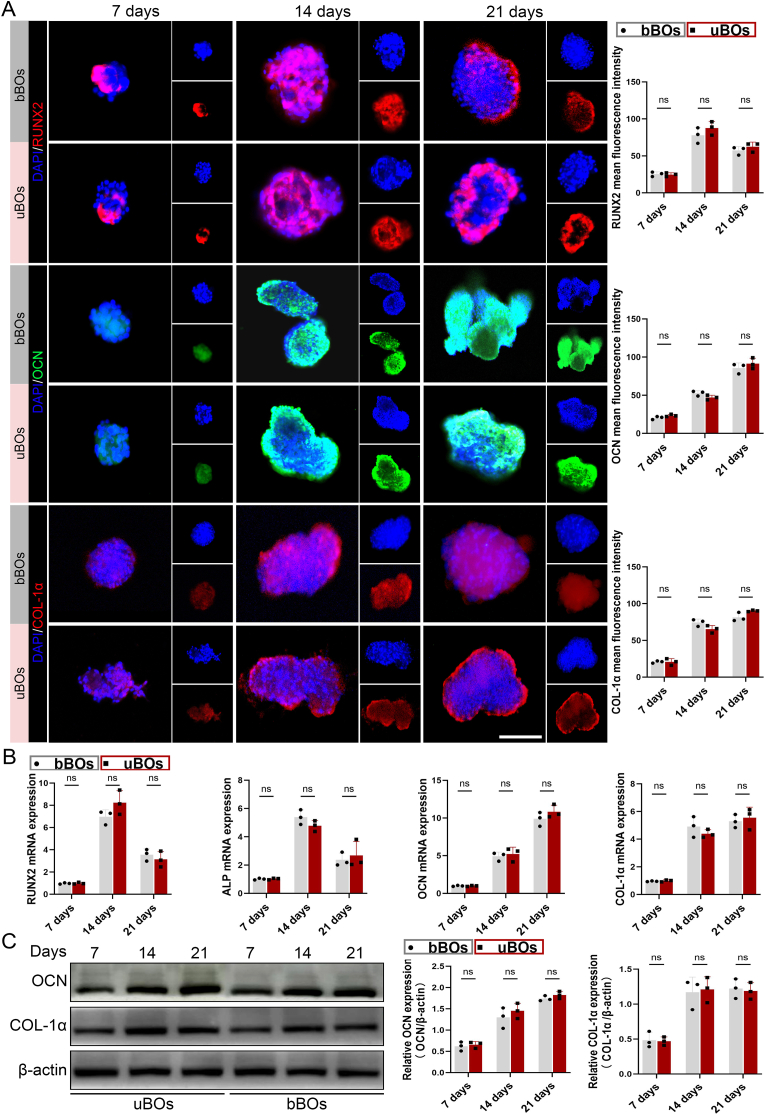
**Rapid generation of uBOs and its characteristics evaluation.** (A) On the 7th, 14th, and 21st days of directed osteogenic induction, immunofluorescence was used to detect the expression of early osteogenic marker *RUNX2*, mid-stage osteogenic marker *COL-1α*, and late-stage osteogenic marker *OCN* in uBOs and bBOs, followed by quantitative analysis. Scale bar = 50 μm. (B) qRT-PCR was conducted to evaluate the mRNA expression levels of osteogenic genes (*RUNX2*, *COL-1α*, *OCN*) in uBOs and bBOs. (C) WB analysis was carried out to assess the protein expression levels of osteogenic markers (*RUNX2*, *COL-1α*, *OCN*) in uBOs and bBOs. Data are presented as mean ± SD (n = 3). p-values are calculated using unpaired *t*-test. “ns” indicates no significance.

Under the stimulation of osteogenic induction medium, USCs@DBM-MPs underwent differentiation and transformation into novel BOs (uBOs). Additionally, bBOs generated from BMSCs and DBM-MPs were used as controls to systematically evaluate the ossification potential of uBOs. Firstly, as time progressed, the results of immunofluorescence staining and quantitative analysis demonstrated that the expression of the early osteogenic marker *RUNX2* in uBOs and bBOs peaked at day 14 and subsequently declined by day 21. However, no significant difference was observed in *RUNX2* expression between uBOs and bBOs. Secondly, the fluorescence analysis of the mid-term osteogenic marker *COL-1α* revealed that the expression levels in uBOs and bBOs were comparable at days 7, 14, and 21, with no discernible differences. Notably, *COL-1α* expression was higher in both groups at days 14 and 21 compared to day 7. Lastly, the fluorescence results for the late osteogenic marker *OCN* indicated that *OCN* expression in uBOs and bBOs progressively increased over time and reached a peak at day 21. Consistent with the other markers, no significant difference in *OCN* expression was detected between uBOs and bBOs at any time point (Fig. 4A). Further qRT-PCR analysis produced consistent results. The expression levels of osteogenic genes (*RUNX2*, *COL-1α*, *OCN*) in uBOs and bBOs were comparable at each time point, showing no statistically significant differences. Additionally, their temporal variation trends aligned with the immunofluorescence findings (Fig. 4B). The Western Blotting (WB) results demonstrated that the protein expression levels of *COL-1α* in uBOs and bBOs progressively increased from day 7 to day 14, with the expression level on day 21 remaining comparable to that on day 14. In contrast, the expression of *OCN* exhibited a gradual increase over time, peaking on day 21. Semi-quantitative analysis revealed that the protein expression levels in uBOs and bBOs at each time point were similar, showing no statistically significant differences (Fig. 4C). These findings indicate that uBOs and bBOs possess comparable osteogenic potential. However, USCs have the characteristics of a wider source, complete non-invasiveness, and easy to obtain. Therefore, uBOs may represent an organoid strategy that is more conducive to promotion and translation. Additionally, based on the temporal detection of the aforementioned osteogenic markers, we propose that the 14th day is the osteogenic active period of uBOs. Therefore, we selected the uBOs at this timepoint for subsequent in-vivo experiments.

### 2.5 Explore the in-vitro generation mechanism of uBOs

**Fig. 5:**
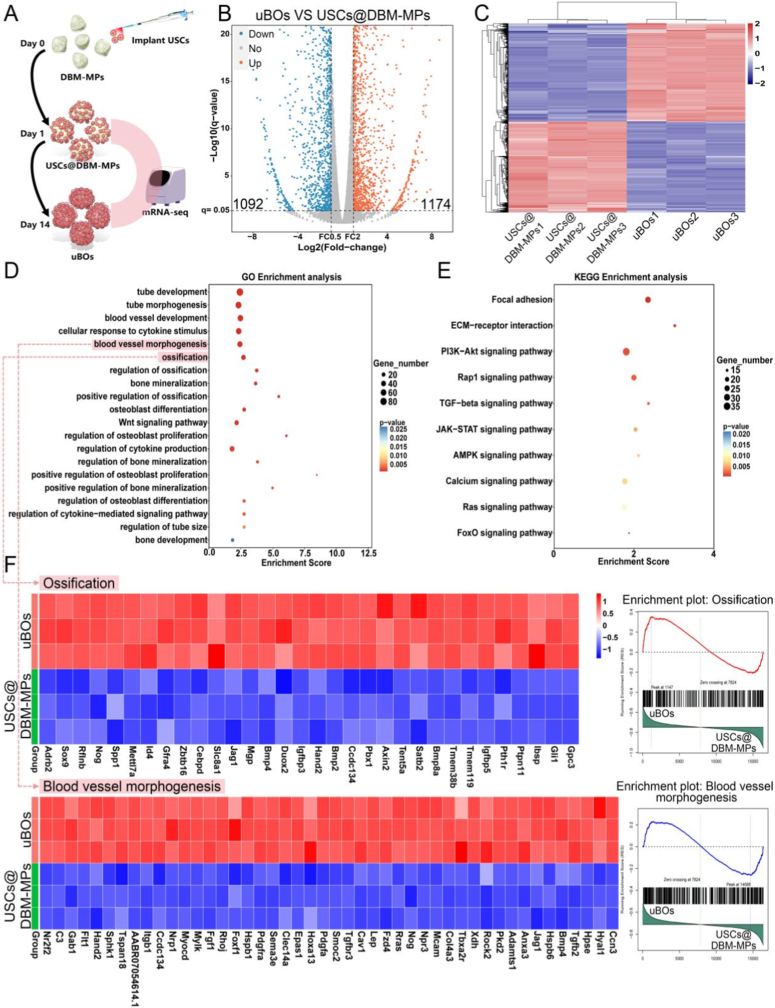
**mRNA-seq reveals the *in vitro* formation mechanism of uBOs.** (A) Schematic diagram of the research design. (B) Volcano plot depicting DEGs profiles in uBOs compared to USCs@DBM-MPs. (C) Heatmap analysis of the DEGs. (D) GO enrichment analysis of osteogenic and angiogenic related biological processes based on upregulated DEGs in uBOs. (E) KEGG analysis of osteogenic and angiogenic related signaling pathways using the upregulated DEGs in uBOs. (F) Heatmap analysis and GSEA analysis of ossification and blood vessel morphogenesis.

To explore the generation mechanism of uBOs, we extracted the RNA of uBOs for transcriptome sequencing, and used USCs@DBM-MPs that had just been inoculated with stem cells as the control (Fig. 5A). The volcano plot and heatmap analysis of differentially expressed genes (DEGs) showed that compared with the USCs@DBM-MPs group, 1174 genes in the uBOs group were upregulated, and 1092 genes were downregulated (Fig. 5B and C). Further Gene Ontology (GO) analysis of the upregulated genes in uBOs revealed that uBOs were highly enriched in osteogenic and angiogenic-related biological processes, including “tube development”, “tube morphogenesis”, “blood vessel morphogenesis”, “ossification”, “bone mineralization”, “bone development”, etc. (Fig. 5D). Additionally, Kyoto Encyclopedia of Genes and Genomes (KEGG) analysis of the upregulated genes in uBOs also showed that uBOs were enriched in some classic osteogenic and angiogenic signaling pathways, including “*TGF-beta* signaling pathway”, “*PI3K-Akt* signaling pathway”, “*Ras* signaling pathway”, etc. (Fig. 5E). Therefore, we considered that many biological processes and signaling pathways related to osteogenic and angiogenic processes might be involved in the generation of uBOs, and this biological effect would be very beneficial for remodeling the bone microenvironment. To further verify this, we selected the gene sets of “ossification” and “blood vessel morphogenesis” for heatmap visualization. It can be seen that osteogenic genes (*BMP-2*, *SPP1*, *BMP-4*, *BMP-8a*, etc.) and angiogenic genes (*Hspb1*, *Pdgfa*, *Sema3e*, *Flt1*, etc.) were significantly upregulated in uBOs. Consistent with Gene Set Enrichment Analysis (GSEA), these data indicate that numerous factors promoting osteogenesis and angiogenesis are overexpressed during uBOs formation (Fig. 5F).

### 2.6 uBOs can further promote angiogenesis and ossification via the paracrine mechanism

**Fig. 6:**
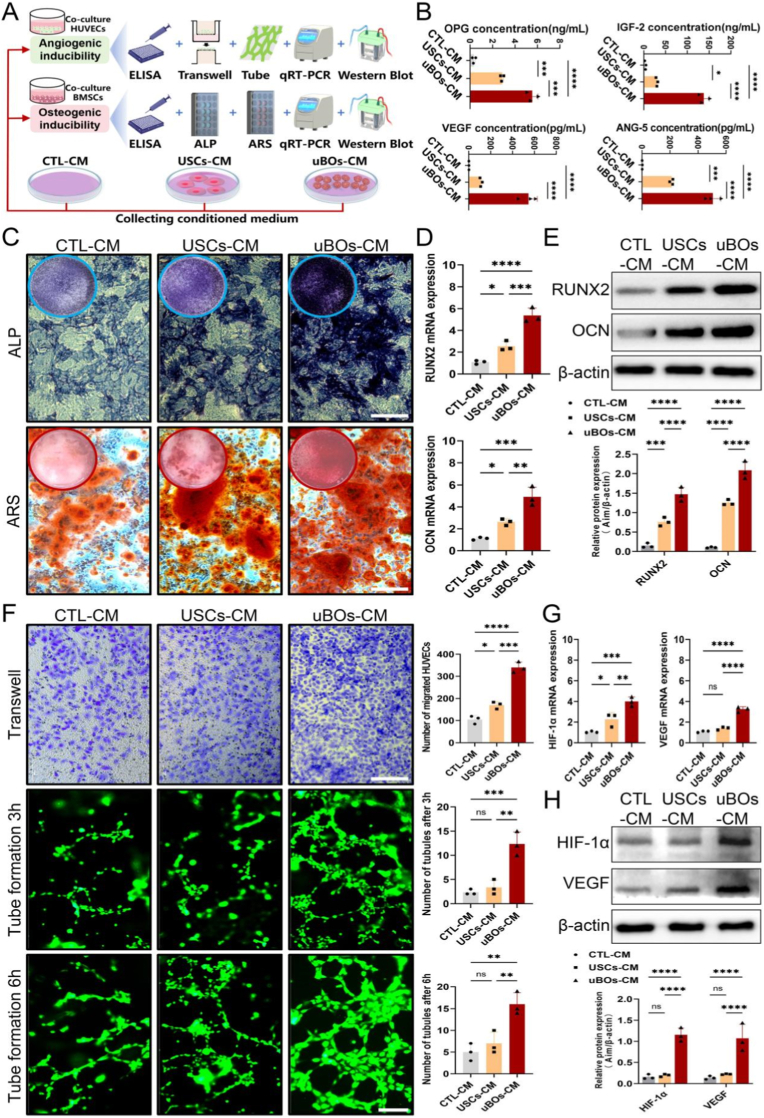
**uBOs can stimulate osteogenesis and angiogenesis through paracrine mechanisms.** (A) Schematic diagram of the research design. (B) ELISA was performed to quantify osteogenic cytokines (*OPG*, *IGF-2*) and angiogenic cytokines (*VEGF*, *ANG-5*) in uBOs-CM, USCs-CM, and CTL-CM. (C) ALP and ARS staining showed the stimulating effects of uBOs-CM, USCs-CM, and CTL-CM on the osteogenic differentiation of BMSCs. Scale bar = 250 μm. (D, E) The expression levels of osteogenic markers (*RUNX2*, *OCN*) were assessed by qRT-PCR and WB in each group. (F) Transwell and tube formation assays showed the chemotactic and tube formation stimulating effects of uBOs-CM, USCs-CM, and CTL-CM on HUVECs. Scale bar = 200 μm. (G, H) The expression levels of angiogenic markers (*HIF-1α*, *VEGF*) were evaluated by qRT-PCR and WB in each group. Data are presented as mean ± SD (n = 3). p-values are calculated using one-way analysis of variance (ANOVA) with Bonferroni post hoc test. ∗p < 0.05, ∗∗p < 0.01, ∗∗∗p < 0.001, ∗∗∗∗p < 0.0001, and “ns” indicates no significance.

It has been revealed by previous transcriptomic sequencing results that uBOs secrete molecules associated with osteogenesis and angiogenesis during the development process. This may represent the biological mechanism through which uBOs indirectly promote bone regeneration. Consequently, this section presents an experimental validation of this paracrine mechanism, We extracted the uBOs-conditioned medium (uBOs-CM), the USCs-conditioned medium (USCs-CM), and the control-conditioned medium (CTL-CM) for testing. These three types of conditioned medium were then co-cultured with BMSCs and HUVECs to evaluate their effects on osteogenic differentiation and angiogenesis (Fig. 6A). First, ELISA results demonstrated that compared to the CTL-CM group and USCs-CM group, the secretion levels of osteogenic factors *OPG* and *IGF-2* in the supernatant of the uBOs-CM group were significantly elevated. Meanwhile, the secretion of angiogenic factors *VEGF* and *ANG-5* was also highest in the uBOs group (Fig. 6B).

In terms of paracrine osteogenic regulation, the ALP and ARS staining results demonstrated that the uBOs-CM significantly enhanced the osteogenic differentiation potential of stem cells, as evidenced by increased ALP activity and calcium nodule deposition (Fig. 6C). Consistently, qRT-PCR and WB analyses revealed that the expression levels of osteogenic-related molecules *OCN* and *RUNX2* were markedly upregulated in stem cells cultured under the uBOs-CM, surpassing those observed in stem cells cultured with the USCs-CM group and CTL-CM group (Fig. 6D and E). This indicates that uBOs can further enhance the osteogenic properties of stem cells through the paracrine mechanism.

In terms of paracrine angiogenic regulation, the transwell assay demonstrated that the number of migrated HUVECs significantly increased when induced by the uBOs-CM (Fig. 6F). Moreover, the tube formation assay indicated that under the stimulation of the uBOs-CM, HUVECs formed more extensive and well-organized capillary-like network structures. In contrast, HUVECs treated with the USCs-CM and the CTL-CM exhibited discontinuous branching patterns in the tube wall structures (Fig. 6F). Further qRT-PCR and WB analyses revealed that HUVECs cultured in uBOs-CM significantly upregulated the expression of angiogenesis-related molecules, including *VEGF* and *HIF-1α* (Fig. 6G and H). These findings also indicate that uBOs can enhancing the angiogenic properties of HUVECs via the paracrine mechanism.

### 2.7 uBOs have the ability of autonomous osteogenesis in the subcutaneous tissue of nude mice

**Fig. 7:**
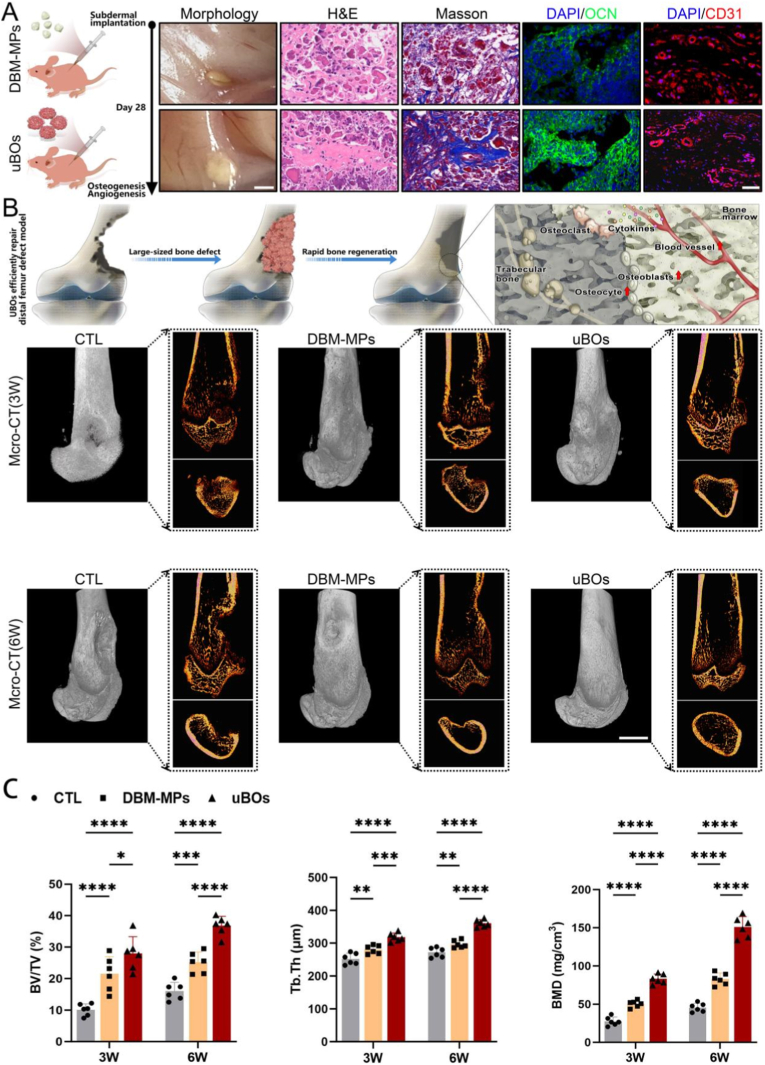
**uBOs possess the capability to autonomously form bone and effectively repair critical-sized bone defects in-vivo.** (A) uBOs were subcutaneously injected into nude mice, and histological evaluation was performed 4 weeks later. Scale bar = 1.5 mm or 50 μm. (B) 3 weeks and 6 weeks after femoral distal bone defect modeling, representative three-dimensional CT reconstructions and two-dimensional morphological images of femoral distal bone defects were obtained. Scale bar = 3 mm. (C) Statistical analysis was carried out to compare BV/TV, Tb.Th, and BMD values of newly formed bone among the three groups. Data are presented as mean ± SD (n = 6). p-values are calculated using one-way ANOVA with Bonferroni post hoc test. ∗p < 0.05, ∗∗p < 0.01, ∗∗∗p < 0.001, ∗∗∗∗p < 0.0001.

DBM-MPs or uBOs were subcutaneously implanted into nude mice to evaluate their autonomous osteogenic capabilities. As shown in Fig. 7A, 28 days post-transplantation, signs of bone fusion for uBOs were observed in the subcutaneous tissue of nude mice, whereas DBM-MPs remained as discrete bone particles. Further histological staining with H&E and Masson demonstrated the formation of immature bone trabeculae in the subcutaneous tissue of nude mice in the uBOs group. In contrast, in the DBM-MPs implantation group, only isolated bone particles were stained, with no evident signs of new bone formation. Immunofluorescence staining results confirmed that the expression level of the late osteogenic marker protein *OCN* was significantly higher in the uBOs group compared to the DBM-MPs group. Moreover, the number of microvessels labeled by *CD31* was markedly increased in the uBOs group relative to the DBM-MPs group. Meanwhile, qRT-PCR analysis revealed elevated transcriptional levels of osteogenesis-related genes *COL-1α* and *OCN* in the uBOs group. Similarly, the expression levels of angiogenesis-related genes *PDGF-BB* and *VEGF* were also significantly higher in the uBOs group than in the DBM-MPs group (Fig. S2). Collectively, these findings indicate that even in the subcutaneous environment of nude mice, uBOs possess the capacity to spontaneously fuse and form bone trabecular structures while inducing the neogenesis of microvessels.

### 2.8 In-situ injection of uBOs rapidly repairing bone defect

In this in-vivo study, rats with cylindrical defects in the distal femur were randomly assigned to three treatment groups: the PBS injection group (CTL group), the DBM-MPs injection group (DBM-MPs group), and the uBOs injection group (uBOs group). As illustrated in Fig. 7B, three-dimensional CT reconstruction images and two-dimensional cross-sectional images demonstrated that at 3 weeks post-operation, only a small amount of scattered and irregular bone formation was observed at the defect edge in the CTL group. In contrast, both the DBM-MPs group and the uBOs group exhibited evident new bone formation around the defect site, which grew inward and significantly reduced the defect area. Notably, the uBOs group showed more extensive new bone formation and a smaller residual defect area compared to the DBM-MPs group. At 6 weeks post-operation, the CTL group exhibited some new bone formation, but the defect area remained substantial. The DBM-MPs group formed cortical bone to a certain extent, however, it was relatively sparse, leaving a circular bone defect depression. Conversely, the uBOs group displayed markedly greater new bone volume than the other two groups, the newly formed bone nearly covered the entire defect site, forming continuous and relatively complete cortical bone with almost no discernible defect, indicating the most effective bone repair capability among the three groups. Statistical analysis further revealed that at 3 and 6 weeks post-operation, the bone volume fraction (BV/TV), trabecular thickness (Tb.Th), and bone mineral density (BMD) in the uBOs group were significantly higher than those in the DBM-MPs group and the CTL group (Fig. 7C).

**Fig. 8:**
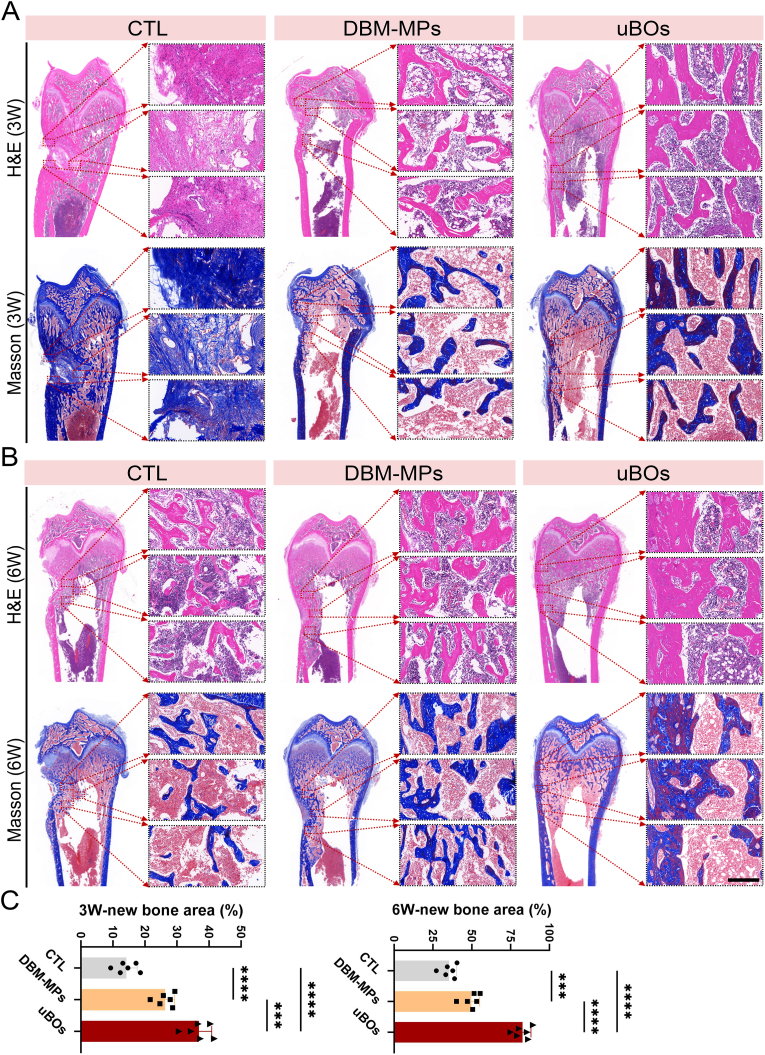
**Histological evaluation of uBOs injection for repairing femoral distal bone defects.** (A) After 3 weeks of modeling, H&E staining and Masson staining of the femoral distal bone defect sections of each group. (B) After 6 weeks of modeling, H&E staining and Masson staining of the femoral distal bone defect sections of each group. Scale bar = 250 μm. (C) Semi-quantitative analysis of new bone formation in the postoperative bone defect area. Data are presented as mean ± SD (n = 6). p-values are calculated using one-way ANOVA with Bonferroni post hoc test. ∗∗∗p < 0.001, ∗∗∗∗p < 0.0001.

Moreover, H&E and Masson staining further corroborated the Micro-CT results. The staining results revealed that in the 3rd week post-operation, no evident new bone formation was observed in the CTL group, while a substantial amount of fibrous-like tissue was present in the defect area. In contrast, both the DBM-MPs group and the uBOs group exhibited a certain degree of new bone formation, but the healing of the uBOs group was better than that of the DBM-MPs group, and the uBOs group had more new bone trabeculae (Fig. 8A). By the 6th week post-operation, the uBOs group showed a significant increase in new bone formation, characterized by relatively dense lamellar bone that completely covered the defect area. Conversely, prominent bone defect depressions were still evident in both the DBM-MPs group and the CTL group (Fig. 8B). Semi-quantitative analysis confirmed that the proportion of new bone in the uBOs group was significantly higher than in the DBM-MPs group and the CTL group (Fig. 8C).

### 2.9 uBOs not only directly fill and repair bone defects, but also positively stimulate the coupling mechanism of osteogenesis and angiogenesis via the paracrine pathway

**Fig. 9:**
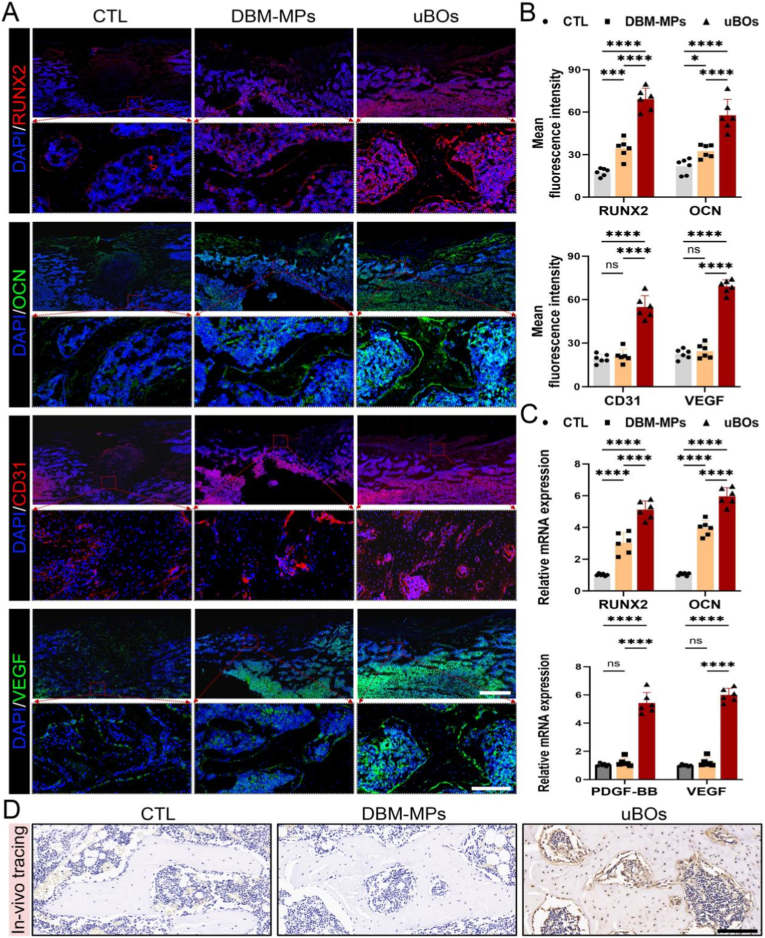
**Evaluation of the in-vivo biological functions of uBOs and its in-vivo tracing.** (A, B) After 3 weeks of modeling, immunofluorescence staining was employed to assess the expression levels of osteogenic markers (*RUNX2*, *OCN*) and angiogenic markers (*CD31*, *VEGF*), and semi-quantitative analysis was performed. Scale bar = 1 mm or 250 μm. (C) qRT-PCR was used to quantitatively detect the gene expression levels of osteogenic markers (*RUNX2*, *OCN*) and angiogenic markers (*PDGF-BB*, *VEGF*) in newly formed bone tissue. (D) After 6 weeks of implantation of uBOs, in-vivo cell tracking was performed using immunohistochemistry. In the uBOs group, more human nuclei-stained cells were detected within the bone trabeculae. Scale bar = 200 μm. Data are presented as mean ± SD (n = 6). p-values are calculated using one-way ANOVA with Bonferroni post hoc test. ∗p < 0.05, ∗∗∗p < 0.001, ∗∗∗∗p < 0.0001, and “ns” indicates no significance.

As previously confirmed by in-vitro experiments, uBOs can further enhance the coupling of osteogenesis and angiogenesis via paracrine mechanisms. Therefore, it is essential to revalidate this effect *in vivo*. At 3 weeks post-operation, immunofluorescence analysis revealed that the expressions of *RUNX2* and *OCN* were significantly higher in the uBOs group compared to other groups, followed by the DBM-MPs group, while the fluorescence intensity in the CTL group was the lowest. Additionally, *CD31* immunofluorescence results demonstrated that the uBOs group exhibited the greatest number of newly formed blood vessels and the highest expression of the angiogenic protein *VEGF* (Fig. 9A and B). These findings were corroborated by qRT-PCR results (Fig. 9C), which showed consistent trends. Among all groups, the uBOs group displayed the highest expression levels of osteogenic markers (*RUNX2*, *OCN*) and angiogenic markers (*PDGF-BB*, *VEGF*). Collectively, these results indicate that uBOs can indeed induce angiogenesis and immigration in the defect area, and couple with the osteogenesis process to promote endogenous bone regeneration.

At 6 weeks post-operation, immunohistochemical staining was performed to detect anti-human nuclear antibodies in the newly formed bone regions of each group. A significant number of positive cells were observed in the bone regeneration area of the uBOs group, with a higher density of positive osteocytes evident within the new bone trabeculae. In contrast, minimal to no positive expression was detected in or around the bone trabeculae of the DBM-MPs group and the CTL group (Fig. 9D). These findings suggest that uBOs retained robust biological activity throughout the *in vivo* bone repair process.

## 3 Discussion

Bone is a highly sophisticated connective tissue organ [4,38]. Natural bone tissue possesses a certain degree of self-healing capability [6]. However, in cases of large-area bone defects caused by trauma, infection, tumors, etc., namely critical-sized bone defects, the bone tissue lacks sufficient self-regeneration capacity and often necessitates bone graft intervention [39,40]. Currently, autologous bone grafting is regarded as the gold standard for treating bone defects. Nevertheless, its source is limited, it cannot be arbitrarily cut or shaped, and it inevitably poses risks such as pain, infection, and bleeding at the donor site, thereby severely restricting its application [5,41]. Allogeneic and xenogeneic bone grafting avoid the risks associated with donor-site sacrifice but exhibit weak osteoconductivity and osteoinductivity, inevitable immune rejection, and potential cross-infection or disease transmission [12,42]. Consequently, bone tissue engineering has emerged as a current research focus in addressing bone defects. Among the advancements in this field, various novel bone substitutes, including 3D-printed scaffolds, injectable hydrogels, nano-microspheres, and bioceramics, have been developed and demonstrated significant research value. Regrettably, these substitutes struggle to replicate the complex biological structure of natural bone tissue, making it challenging to achieve efficient primary bone healing [43,44].

Recently, the emergence and development of organoid technology have provided novel insights into achieving efficient bone tissue regeneration [9]. To date, related research has successfully cultivated first-generation organoids for various tissues, including bone, cartilage, intestine, brain, heart, etc [15,45,46]. BOs represent an emerging field focused on replicating the complex structure and multifaceted functions of bone tissue in-vitro, enabling their direct application as bone replacement units in-vivo [31]. However, cultivating BOs remains a highly challenging endeavor, heavily reliant on the design and construction of three-dimensional carriers as well as the use of stem cells [47,48]. In the past, three-dimensional carriers fabricated from bioactive materials have been combined with seeded stem cells to form microtissues mimicking the spatial architecture of bone through directional induction and three-dimensional culture techniques [49]. Consequently, the selection and construction of biological three-dimensional carriers are critical, as they must not only accurately replicate the composition of the natural bone matrix, but also support and guide cell growth and differentiation. In previous studies, scholars frequently employed GelMA microspheres, matrix gel, calcium phosphate, and polymer synthetic scaffolds as cultivation carriers for BOs [15,50,51]. These approaches have successfully enabled the in-vitro cultivation of BOs and yielded promising outcomes in in-vivo bone defect models. Nevertheless, from a theoretical perspective, the aforementioned three-dimensional carriers exhibit certain discrepancies compared to the organic and inorganic components of native bone matrices, limiting their biomimetic fidelity and thereby affecting the high similarity of the constructed BOs [[52], [53], [54]]. Hence, in this study, we successfully prepared DBM using a self-developed decellularization system. Subsequently, DBM was innovatively processed into a novel carrier—DBM-MPs, via freeze-grinding and filtration techniques. Experimental verification confirmed that cellular components were entirely removed from DBM-MPs, ensuring excellent biocompatibility. Moreover, the collagen and calcium-phosphorus compounds of the primary tissue were fully preserved within DBM-MPs, which exhibited high similarity to natural bone tissue and effectively promoted the osteogenic differentiation of stem cells. Interestingly, in the in-vivo bone defect model, compared with the CTL group, the DBM-MPs group induced more new bone and had a smaller defect area. In summary, the DBM-MPs developed in this study may serve as an ideal three-dimensional carrier for constructing BOs.

In the selection of stem cells for constructing BOs. Most scholars frequently utilize classic BMSCs, induced pluripotent stem cells, adipose-derived stem cells, embryonic stem cells, and periosteal stem cells as seed cells for cultivating BOs [48,[55], [56], [57]]. Currently, researchers typically load these seed cells onto culture scaffolds and induce their directed differentiation into osteoblasts to reconstruct the bone formation unit. Consequently, seed cells not only preserve the genotype of the donor but also exhibit high self-renewal capacity and multi-lineage differentiation potential [58,59]. Unfortunately, the acquisition of the aforementioned seed cells typically necessitates invasive procedures and is constrained by limited sources [60]. From the standpoint of subsequent clinical translation, this limitation hinders large-scale production and thus makes it challenging to fulfill the treatment demands of the growing number of patients with bone defects. To address these issues, our study innovatively identified USCs as a promising alternative seed cell source for BOs. USCs are derived from human waste urine, thereby offering a non-invasive, abundant, and sustainable resource for tissue engineering [61,62]. Importantly, in-vitro experiments confirmed that USCs exhibit comparable osteogenic performance to BMSCs. Furthermore, when USCs or BMSCs were respectively loaded onto DBM-MPs for directional induction to form uBOs or bBOs, various molecular biology analyses demonstrated that uBOs and bBOs possess similar bone-inducing functions without statistically significant differences. Notably, uBOs performed admirably in an in-vivo femoral condyle bone defect model, achieving substantial bone regeneration and complete defect coverage by the sixth week. In summary, USCs represent a superior non-invasive seed cell source, and uBOs co-cultured by USCs and DBM-MPs could constitute a novel type of bone organoid capable of achieving efficient bone regeneration, non-invasive procurement, and a sustainable supply.

Furthermore, the research on the regeneration mechanism of BOs remains in still in the initial exploration stage [63,64]. In our study, transcriptomics analysis revealed that uBOs were significantly enriched in biological processes such as “ossification,” “bone development,” “tube formation,” and “cytokine interaction” during their development. This suggests that during the generation of uBOs, not only does self-ossification occur, but there may also be secretion of active factors associated with bone development and angiogenesis [65,66]. Based on this, we performed additional experimental validation. By collecting the supernatant during the development of uBOs, we demonstrated that the uBOs-CM significantly stimulated the migration and tube formation of HUVECs, as well as upregulated the expression of angiogenic molecules. These findings suggest that uBOs may promote angiogenesis via a paracrine mechanism. This was further corroborated in subsequent in-vivo experiments. In-vivo immunofluorescence staining revealed a significant increase in the number of microvessels labeled by *CD31* in the uBOs group, with the angiogenic protein *VEGF* exhibiting high expression levels. Moreover, our study uncovered that the uBOs-CM could enhance the osteogenic differentiation and mineralization of stem cells, while upregulating the expression of osteogenic-related proteins. This indicates that uBOs can also indirectly augment the osteogenic potential of stem cells through paracrine signaling. Collectively, the paracrine mechanism of uBOs elucidated in our study may represent a positive feedback loop during rapid bone regeneration, facilitating the coupling mechanism between osteogenesis and angiogenesis.

All in all, in this study, novel bone organoid—uBOs were successfully cultivated *in vitro* based on USCs and DBM-MPs. and the paracrine mechanisms underlying angiogenesis stimulation and osteogenesis promotion were preliminarily elucidated. Notably, prior to implantation into the rat femoral condyle bone defect model, uBOs underwent osteogenic-directed cultivation *in vitro*, forming a significant amount of extracellular matrix components and establishing tight connections among various cellular matrices. As early as 3 weeks post-implantation, uBOs facilitated complete bridging of the defect, accompanied by mineralized tissue filling and new bone remodeling. By 6 weeks, the general structure of the original long bone was largely restored, including the formation of denser cortical bone. Thus, these novel uBOs demonstrate rapid bone regeneration capabilities in bone repair applications and hold excellent prospects for future use. Nevertheless, this study has certain limitations, for instance, the KEGG signaling pathway mechanism derived from transcriptomics analysis requires further validation through additional molecular biology experiments. Meanwhile, from the perspective of clinical transformation, further large animal bone defect models are still required to verify the efficient bone regeneration performance of uBOs.

## 4 Conclusions

In this study, DBM was initially prepared using an independent decellularization system. Subsequently, a novel bone organoid carrier—DBM-MPs, characterized by high biomimicry, easy accessibility, and superior osteoinductivity, was constructed via freeze-milling and filtration. USCs with non-invasive, widely available, and sustainable were utilized as the seed cells for bone organoid construction. *In vitro* characterization experiments confirmed that USCs exhibit osteogenic potential comparable to BMSCs. Furthermore, USCs were loaded onto DBM-MPs for osteogenic directional induction, and a novel bone organoid—uBOs, was successfully generated within 14 days. These uBOs demonstrate paracrine functions capable of stimulating angiogenesis and osteogenesis. In a rat femoral condyle defect model, minimally invasive injection of uBOs into the bone defect area achieved rapid bone regeneration within only 6 weeks, perfectly repairing the defect area. Consequently, uBOs can serve not only as a bone substitute unit for direct filling and repair of bone defects, but also continuously induce angiogenesis and bone fusion at the defect site through their paracrine mechanism, offering a brand-new and efficient tissue engineering strategy for bone defect treatment.

## 5 Methods

### 5.1 Ethics statement

This study has been approved by the Ethics Committee of the Xiangya Hospital, Central South University, China. The *in vivo* experiments involving F344 rats and nude mice have also been approved by the Institutional Animal Care and Use Committee of Central South University, China.

### 5.2 Preparation of DBM-MPs

The cancellous bone was harvested from pig vertebrae obtained at a local slaughterhouse and cut into small fragments. These fragments were wrapped in gauze and immersed in a cell-decellularization solution consisting of 0.1 % Triton X-100 (Beyotime, China) and 2 % SDS (Aladdin, China), followed by vigorous stirring for 12 h. Afterward, the bone fragments were rinsed three times with pre-cooled PBS, each rinse lasting 8 h. Subsequently, the samples were transferred into a ribozyme solution containing 500 U/ml type I DNase and 1 mg/ml RNase (Sigma-Aldrich, USA), maintained at 37 °C, and stirred for an additional 12 h. The bone fragments were washed three times with PBS (each time for 8 h) again, and then placed in the vacuum freeze-drying machine (FD8-5T, SIM, Newark, NJ) for freeze-drying to obtain DBM. Subsequently, the DBM blocks were frozen ground using a low-temperature grinder (Servicebio, China) into DBM powder, and then filtered through a steel wire mesh with a pore size of 80 μm to obtain DBM-MPs.

### 5.3 Evaluation of DBM-MPs

After fixing DBM with 4 % paraformaldehyde for 48 h, standard decalcification procedures were carried out. Subsequently, paraffin-embedded tissue sections were prepared for histological analysis. Natural bone was used as the control group. H&E, Goldner, and DAPI staining techniques were applied to assess the presence of residual cellular components and nuclear material within the DBM. Furthermore, DAPI/*COL-1α* (66761-1-lg, Proteintech) immunofluorescence labeling and SR staining were conducted to evaluate the preservation of extracellular matrix proteins in the DBM. SEM (Mira4 LMH, TESCAN, Czech Republic) combined with EDS was employed to examine the microstructural characteristics and elemental composition (calcium and phosphorus) of the bone matrix before and after the decellularization process. The DNA content in DBM-MPs was quantified using the PicoGreen DNA assay kit (Invitrogen, USA). Additionally, the particle size distribution of DBM-MPs was analyzed using a laser diffraction particle size analyzer (Mastersizer 2000, UK). A subcutaneous DBM-MPs injection model was established in nude mice. Briefly, DBM-MPs were mixed with fluorescein-Cy5 under light-protected conditions, and the suspension (80 mg of microparticles in 0.2 mL PBS) was administered subcutaneously using a 1 mL syringe. Fluorescence imaging signals of DBM-MPs in the subcutaneous region were then monitored immediately post-injection, on day 3, and on day 5 using a small animal *in vivo* fluorescence imaging system (AniView100, China).

### 5.4 Isolation and culture of BMSCs

Human BMSCs were isolated from bone marrow aspirates obtained from the anterior iliac crest of donors. Primary BMSCs were subsequently harvested through centrifugation. The isolated BMSCs were then routinely expanded and cultured in α-MEM (Gibico, USA) supplemented with 10 % FBS (Gibico, USA) and 1 % double antibiotic solution (Gibico, USA) under standard cell culture conditions (37 °C, 5 % CO_2_).

### 5.5 Biocompatibility of DBM-MPs

The 3rd generation BMSCs were seeded at a density of 2.5 × 10^4^ cells per well in the bottom of a 48-well plate. Then, 10 mg of DBM-MPs was added to the culture system. At the 1st, 4th, and 7th days of co-culture, CCK-8 assay (Beyotime, China) was used to incubate the well plates at each time point, and the OD450 absorbance values were recorded. Proliferation curves were then generated based on these measurements.

In addition, the co-culture system of RAW 264.7 cells and DBM-MPs was established using the same method. A blank control CTL group and a positive control LPS group were set up. At the 1st, 4th, and 7th days of co-culture, the cell viability and morphology were evaluated by live/dead staining (BestBio, China) and DAPI/phalloidin (Yeasen, China) fluorescence staining. At the 4th day, the cell supernatants were collected, and the secretion levels of *TNF-α*, *IL-6*, and *IL-1β* in each group were determined according to the instructions of the ELISA kit (Elabscience or Cusabio, China).

### 5.6 Osteogenic inducibility of DBM-MPs

ALP and ARS staining were employed to assess the stimulatory effect of DBM-MPs on the osteogenic differentiation of BMSCs. After seeding BMSCs into 24-well plates and culturing for 24 h, the medium was replaced with osteogenic induction medium (Starfish Biotechnology, China) and DBM-MPs were added simultaneously. After osteogenic directional induction for 14 days, the cells were fixed with 4 % paraformaldehyde, washed with PBS, and stained with the ALP chromogenic kit (Beyotime, China). After 21 days of osteogenic directional induction, the calcium nodules were stained with the ARS solution (Starfish Biotechnology, China), and representative images were captured using a digital camera and microscope. Furthermore, qRT-PCR analysis was conducted on day 14 to evaluate the expression of osteogenic markers (*RUNX2*, *COL-1α*, *OCN*) in each group. In simple terms, total RNA was extracted using Trizol reagent (CW Biotech, China), and cDNA was synthesized using the reverse transcription kit (Takara, Japan). The cDNA was amplified in a qPCR system in a 20 μL reaction mixture. GAPDH was used as a reference for data processing. The primer sequences are shown in Table S1.

### 5.7 Isolation and culture of USCs

The human USCs required for the experiment were obtained from clean midstream urine. The collected urine was centrifuged at 1000 r/min for 10 min at 4 °C, and the supernatant was carefully removed. The precipitated cells were resuspended in PBS and re-centrifuged for washing. After washing, the bottom cell precipitate was collected, resuspended in α-MEM medium (Gibico, USA) supplemented with 10 % FBS (Gibico, USA) and 1 % penicillin-streptomycin solution (Gibico, USA), and inoculated into cell culture flasks and placed in a constant temperature incubator at 37 °C with 5 % carbon dioxide for routine expansion and culture.

### 5.8 Flow cytometry analysis

Flow cytometry was conducted using a BD FACS Aria III instrument, and data were analyzed with FlowJo software (version 10.8.0). To identify the surface marker profile of USCs, the following flow cytometry antibodies were used: *CD90* (328109, BioLegend), *CD73* (344005, BioLegend), and *CD29* (303007, BioLegend) as positive markers; and *CD34* (378605, BioLegend), *CD31* (989002, BioLegend), and *CD45* (982316, BioLegend) as negative markers.

### 5.9 Osteogenic potential of USCs and BMSCs

Digest and resuspend the 3rd generation USCs and BMSCs, and seeded at a density of 10 × 10^4^ cells per well into a 24-well culture plate. Subsequently, the medium was replaced with osteogenic induction medium. Following routine osteogenic induction for 14 and 21 days, the samples were fixed with 4 % paraformaldehyde. ALP staining was performed using an ALP chromogenic kit (Beyotime, China), while ARS solution (Starfish Biotechnology, China) was conducted to assess calcium nodule formation. Representative images of each well were captured using a digital camera and microscope. Furthermore, total RNA was extracted after 14 days of osteogenic induction, and the mRNA expression levels of key osteogenic markers (*RUNX2*, *COL-1α*, and *OCN*) were quantified using qRT-PCR analysis. The primer sequences are shown in Table S1.

### 5.10 The cell viability, adhesion and proliferation of USCs@DBM-MPs and BMSCs@DBM-MPs

DBM-MPs (100 mg) were placed at the bottom of a 24-well low-adhesion cell culture plate (174930, Thermo Fisher), and inoculate USCs or BMSCs (derived from the same batch of donors) at a cell density of 1.5 × 10^4^ per well. On days 1, 7, and 14 of co-culture. Cell viability in both groups was assessed using optical microscopy combined with live/dead cell staining (BestBio, China). At each time point, cell adhesion was evaluated via DAPI/phalloidin (Yeasen, China) fluorescence staining. Furthermore, the CCK-8 assay (Beyotime, China) was employed to analyze the proliferation of USCs@DBM-MPs and BMSCs@DBM-MPs.

### 5.11 Cultivation and evaluations of uBOs

Wash the DBM-MPs that have been sterilized by ethylene oxide with PBS. Then, place 100 mg of DBM-MPs at the bottom of a 24-well low-adhesion cell culture plate (174930, Thermo Fisher). Seed USCs or BMSCs (derived from the same batch of donors) at a density of 1.5 × 10^4^ cells per well. After 24 h, replace with osteogenic induction medium (Starfish Biotechnology, China) and continue induction for 7 days, 14 days, and 21 days to obtain uBOs and bBOs (as controls). Fix the samples at the corresponding time points with 4 % paraformaldehyde, permeabilize the samples with 0.1 % Triton X-100 for 10 min, add ready-to-use goat serum to cover the samples for 45 min, incubate with specific primary antibodies overnight, then use fluorescent secondary antibodies for labeling, wash with PBS, and perform DAPI staining. Finally, observe and evaluate using a fluorescence confocal microscope. Concurrently, total RNA was extracted from uBOs and bBOs at each time point, and the transcription levels of osteogenic markers (*RUNX2*, *ALP*, *COL-1α*, *OCN*) were quantified using qRT-PCR, The relevant primer sequences are shown in Table S1. Additionally, extract the proteins of uBOs and bBOs at each time point, and detect the protein expression levels of mid-to-late osteogenic markers (*COL-1α*, *OCN*) using the WB analysis. The relevant antibodies used in these experiments are listed as follows: *RUNX2* (20700-1-AP, Proteintech), *COL-1α* (66761-1-lg, Proteintech), *OCN* (16157-1-AP, Proteintech).

### 5.12 Transcriptomic mRNA-seq analysis

The uBOs that underwent osteogenic differentiation for 14 days were designated as the experimental group (n = 3), whereas the USCs@DBM-MPs served as the control group (n = 3). Total RNA was extracted using Trizol reagent and subjected to transcriptome sequencing analysis in order to explore the potential mechanisms underlying uBOs generation. Initially, Sequencing libraries were constructed using the NEBNext Ultra II RNA Library Preparation Kit and subsequently sequenced on the Illumina NovaSeq PE150 platform. The reference genome index was then built using HISAT2 (v2.1.0), and raw gene expression levels were quantified using HTSeq (v0.9.1). Gene expression data were normalized using FPKM. Differential expression analysis was carried out using DESeq (v1.38.3). To investigate functional implications, GO enrichment analysis, KEGG pathway enrichment analysis, and GSEA analysis were sequentially performed.

### 5.13 ELISA assay for the paracrine function of uBOs

A total of three groups were established. CTL-CM group: a blank control group with only osteogenic induction medium; USCs-CM group: the supernatant collected during the osteogenic differentiation of USCs; uBOs-CM group: the supernatant collected during the osteogenic differentiation of uBOs. At day 14 of osteogenic induction, the culture medium of all groups was replaced with serum-free basal medium for a 24-h incubation period. Subsequently, the supernatants were harvested and used to quantify the levels of angiogenic factors (*ANG-5*, *VEGF-C*) and osteogenic factors (*IGF-2*, *OPG*) using ELISA kits (Elabscience or Cusabio, China), following the manufacturer's instructions.

### 5.14 Osteogenic regulation of uBOs-CM

BMSCs were seeded into 24-well plates at a density of 10 × 10^4^ cells per well. Subsequently, CTL-CM, USCs-CM, and uBOs-CM with osteogenic induction medium were used to induce osteogenic differentiation for 14 days. ALP activity in each group was assessed after 14 days of induction using an ALP chromogenic kit (Beyotime, China). When the osteogenic induction reached 21 days, the deposition of calcium nodules in each group was detected using the ARS solution (Starfish Biotechnology, China). On the 14th day, the expression levels of osteogenic-related molecules *RUNX2* (20700-1-AP, Proteintech) and *OCN* (16157-1-AP, Proteintech) were detected using qRT-PCR and WB analysis.

### 5.15 Angiogenic regulation of uBOs-CM

The chemotactic effect of uBOs-CM on HUVECs was evaluated using the Transwell migration assay. HUVECs were pre-cultured with CTL-CM, USCs-CM, or uBOs-CM for one week. Subsequently, 3 × 10^4^ HUVECs were resuspended in FBS-free culture medium and seeded into 24-well Transwell chambers (Corning, USA). After 24 h of incubation, the chambers were fixed with 4 % paraformaldehyde for 15 min. Non-migrated cells on the upper surface of the membrane were removed using cotton swabs. The migrated cells were stained with crystal violet for 10 min and visualized under an inverted microscope to assess migration capacity.

For the tube formation assay, 100 μL of Matrigel (BD Biosciences, USA) was evenly spread onto the bottom of pre-cooled 24-well plates and allowed to solidify at 37 °C for 45 min. HUVECs were then seeded at a density of 8 × 10^4^ cells per well. At 3 and 6 h post-seeding, cells were stained with Calcein-AM (BestBio, China), and the formation of capillary-like structures was examined under a fluorescence microscope (DMI8, Leica, Germany).

Further, qRT-PCR and WB were used to detect the expression of vascularization-related molecules *VEGF* (19003-1-AP, Proteintech) and *HIF-1α* (ab179483, Abcam) in HUVECs stimulated by CTL-CM, USCs-CM or uBOs-CM.

### 5.16 The nude mouse experiment

DBM-MPs or uBOs were subcutaneously injected into 4-week-old nude mice (15 mice per group) to assess their autonomous osteogenic capacity. Following 28 days of post-injection observation, subcutaneous tissue from the injection site was harvested and subjected to standard fixation, paraffin embedding, and sectioning. H&E and Masson staining were performed to evaluate trabecular bone formation by uBOs. Concurrently, immunofluorescence staining with DAPI/*OCN* and DAPI/*CD31* was carried out to assess osteogenic and angiogenic effects in the two experimental groups. In addition, total RNA was extracted from the subcutaneous tissue of nude mice, and the expression levels of osteogenic markers (*COL-1α*, *OCN*) and angiogenic markers (*PDGF-BB*, *VEGF*) were analyzed using qRT-PCR. The primer sequences used are listed in Table S1. The following primary antibodies were used: *CD31* (GB11063-2-100, Servicebio), *OCN* (23418-1-AP, Proteintech).

### 5.17 In-vivo bone defect model and uBOs implantation

A total of 54 SPF-grade male F344 rats (8-week-old) were utilized. All surgical procedures were performed under adequate anesthesia with pentobarbital sodium. The skin on the lateral side of the distal femur of the left hind limb was incised using a sterile surgical blade to fully expose the distal femur. A trephine was then used to create a single cortical bone defect with a diameter of 3 mm in the lateral femoral condyle. Following thorough hemostasis, no further intervention was applied to the CTL group. Using serum-free medium as the delivery medium, the DBM-MPs group and the uBOs group were filled with equal volumes of DBM-MPs and uBOs (about 80 mg), respectively, to completely cover the bone defect area. Postoperative antibiotic treatment was administered for three consecutive days. At 3 weeks and 6 weeks post-surgery, the rats were euthanized and femoral specimens were harvested. Subsequently, femoral samples were subjected to micro-CT, histological, and immunohistochemical analyses.

### 5.18 Statistical analysis

All quantitative data are expressed as mean ± SD, and statistical analyses were performed using GraphPad Prism or SPSS 25 software. Experiments were independently repeated at least three times. For comparisons between two groups, an unpaired *t*-test was employed; for comparisons among more than two groups, a one-way ANOVA followed by Bonferroni's post hoc test was conducted. A p-value less than 0.05 was considered statistically significant (∗p < 0.05, ∗∗p < 0.01, ∗∗∗p < 0.001, ∗∗∗∗p < 0.0001).

## CRediT authorship contribution statement

**Yiting Chen:** Writing – original draft, Visualization, Methodology, Investigation, Formal analysis, Data curation, Conceptualization. **Liang Zhang:** Methodology, Investigation. **Zeyu Li:** Visualization, Investigation. **Xinrun Wang:** Formal analysis. **Jie Liu:** Investigation. **Xianwen Wang:** Resources. **Jiyun Hu:** Visualization. **Guotao Wang:** Investigation. **Qihang Huang:** Methodology, Conceptualization. **Yuhao Yuan:** Writing – review & editing, Writing – original draft, Supervision, Project administration, Funding acquisition, Formal analysis, Data curation, Conceptualization.

## Declaration of competing interest

The authors declare that they have no known competing financial interests or personal relationships that could have appeared to influence the work reported in this paper.

## Data Availability

Data will be made available on request.

## References

[1] Zhou S., Xiao C., Fan L., Yang J., Ge R., Cai M., Yuan K., Li C., Crawford R.W., Xiao Y., Yu P., Deng C., Ning C., Zhou L., Wang Y. (2024). Injectable ultrasound-powered bone-adhesive nanocomposite hydrogel for electrically accelerated irregular bone defect healing. J. Nanobiotechnol..

[2] Han Y., Wu Y., Wang F., Li G., Wang J., Wu X., Deng A., Ren X., Wang X., Gao J., Shi Z., Bai L., Su J. (2024). Heterogeneous DNA hydrogel loaded with Apt02 modified tetrahedral framework nucleic acid accelerated critical-size bone defect repair. Bioact. Mater..

[3] Zhao N., Qin L., Liu Y., Zhai M., Li D. (2024). Improved new bone formation capacity of hyaluronic acid-bone substitute compound in rat calvarial critical size defect. BMC Oral Health.

[4] Martorell-de F.L., Torres-Claramunt R., Sanchez-Soler J.F., Perelli S., Hinarejos P., Monllau J.C. (2025). Patellar bone defect grafting does not reduce anterior knee pain after bone-patellar tendon-bone anterior cruciate ligament reconstruction. Knee Surg. Sports Traumatol. Arthrosc..

[5] Oehme S., Milinkovic D.D., Paolucci A., Krafzick S., Fahy S., Damm P., Winkler T., Jung T., Bartek B. (2025). Autologous bone grafting combined with spheroid-based matrix-induced autologous chondrocyte implantation for osteochondral defects of the knee: good clinical outcomes alongside abnormal postoperative gait patterns. Knee Surg. Sports Traumatol. Arthrosc..

[6] Stavropoulos A., Marcantonio C.C., de Oliveira V., Marcantonio E.J., de Oliveira G. (2023). Fresh-frozen allogeneic bone blocks grafts for alveolar ridge augmentation: biological and clinical aspects. Periodontol.

[7] Hu Y., Zhang Y., Ni C.Y., Chen C.Y., Rao S.S., Yin H., Huang J., Tan Y.J., Wang Z.X., Cao J., Liu Z.Z., Xie P.L., Wu B., Luo J., Xie H. (2020). Human umbilical cord mesenchymal stromal cells-derived extracellular vesicles exert potent bone protective effects by CLEC11A-mediated regulation of bone metabolism. Theranostics.

[8] Kim H.D., Amirthalingam S., Kim S.L., Lee S.S., Rangasamy J., Hwang N.S. (2017). Biomimetic materials and fabrication approaches for bone tissue engineering. Adv. Healthcare Mater..

[9] He J., Zhang X., Xia X., Han M., Li F., Li C., Li Y., Gao D. (2020). Organoid technology for tissue engineering. J. Mol. Cell Biol..

[10] Dutta D., Heo I., Clevers H. (2017). Disease modeling in stem cell-derived 3D organoid systems, trends. Mol. Med..

[11] Wang J., Zhou D., Li R., Sheng S., Li G., Sun Y., Wang P., Mo Y., Liu H., Chen X., Geng Z., Zhang Q., Jing Y., Bai L., Xu K., Su J. (2025). Protocol for engineering bone organoids from mesenchymal stem cells. Bioact. Mater..

[12] Zhang S., Li X., Qi Y., Ma X., Qiao S., Cai H., Zhao B.C., Jiang H.B., Lee E.S. (2021). Comparison of autogenous tooth materials and other bone grafts. Tissue Eng. Regen. Med..

[13] Wu X., Hu Y., Sheng S., Yang H., Li Z., Han Q., Zhang Q., Su J. (2025). DNA-based hydrogels for bone regeneration: a promising tool for bone organoids. Mater. Today Bio.

[14] Zhu M., Zhang H., Zhou Q., Sheng S., Gao Q., Geng Z., Chen X., Lai Y., Jing Y., Xu K., Bai L., Wang G., Wang J., Jiang Y., Su J. (2025). Dynamic GelMA/DNA dual-network hydrogels promote woven bone organoid formation and enhance bone regeneration. Adv. Mater..

[15] Wang J., Wu Y., Li G., Zhou F., Wu X., Wang M., Liu X., Tang H., Bai L., Geng Z., Song P., Shi Z., Ren X., Su J. (2024). Engineering large-scale self-mineralizing bone organoids with bone matrix-inspired hydroxyapatite hybrid bioinks. Adv. Mater..

[16] Olijnik A.A., Rodriguez-Romera A., Wong Z.C., Shen Y., Reyat J.S., Jooss N.J., Rayes J., Psaila B., Khan A.O. (2024). Generating human bone marrow organoids for disease modeling and drug discovery. Nat. Protoc..

[17] Zhang W., Hu J., Huang Y., Wu C., Xie H. (2021). Urine-derived stem cells: applications in skin, bone and articular cartilage repair. Burns Trauma.

[18] Liu G., Sun J., Gong M., Xing F., Wu S., Xiang Z. (2021). Urine-derived stem cells loaded onto a chitosan-optimized biphasic calcium-phosphate scaffold for repairing large segmental bone defects in rabbits. J. Biomed. Mater. Res., Part B.

[19] Xu T., Zhang K., Hu Y., Yang R., Tang J., Fu W. (2024). Comparison of the therapeutic efficacy and autophagy-mediated mechanisms of action of urine-derived and adipose-derived stem cells in osteoarthritis. Am. J. Sports Med..

[20] Gao X., Ruzbarsky J.J., Layne J.E., Xiao X., Huard J. (2024). Stem cells and bone tissue engineering. Life-Basel.

[21] Lu W., Zeng M., Liu W., Ma T., Fan X., Li H., Wang Y., Wang H., Hu Y., Xie J. (2023). Human urine-derived stem cell exosomes delivered via injectable GelMA templated hydrogel accelerate bone regeneration. Mater. Today Bio.

[22] Xing F., Yin H.M., Zhe M., Xie J.C., Duan X., Xu J.Z., Xiang Z., Li Z.M. (2022). Nanotopographical 3D-printed poly(epsilon-caprolactone) scaffolds enhance proliferation and osteogenic differentiation of urine-derived stem cells for bone regeneration. Pharmaceutics.

[23] Abbas T.O., Ali T.A., Uddin S. (2020). Urine as a main effector in urological tissue Engineering-A double-edged sword. Cells.

[24] Zhang Y., Huang S., Zhong W., Chen W., Yao B., Wang X. (2021). 3D organoids derived from the small intestine: an emerging tool for drug transport research. Acta Pharm. Sin. B.

[25] Krammer T., Stuart H.T., Gromberg E., Ishihara K., Cislo D., Melchionda M., Becerril P.F., Wang J., Costantini E., Lehr S., Arbanas L., Hormann A., Neumuller R.A., Elvassore N., Siggia E., Briscoe J., Kicheva A., Tanaka E.M. (2024). Mouse neural tube organoids self-organize floorplate through BMP-mediated cluster competition. Dev. Cell.

[26] Lee S., Burner D.N., Mendoza T.R., Muldong M.T., Arreola C., Wu C.N., Cacalano N.A., Kulidjian A.A., Kane C.J., Jamieson C. (2020). Establishment and analysis of three-dimensional (3D) organoids derived from patient prostate cancer bone metastasis specimens and their xenografts. J. Vis. Exp..

[27] Zhou Z., Cui J., Wu S., Geng Z., Su J. (2022). Silk fibroin-based biomaterials for cartilage/osteochondral repair. Theranostics.

[28] Bustamante-Madrid P., Barbachano A., Albandea-Rodriguez D., Rodriguez-Cobos J., Rodriguez-Salas N., Prieto I., Burgos A., Martinez D.V.J., Real F.X., Gonzalez-Sancho J.M., Larriba M.J., Lafarga M., Munoz A., Fernandez-Barral A. (2024). Vitamin D opposes multilineage cell differentiation induced by notch inhibition and BMP4 pathway activation in human colon organoids. Cell Death Dis..

[29] Urbischek M., Rannikmae H., Foets T., Ravn K., Hyvonen M., de la Roche M. (2019). Organoid culture media formulated with growth factors of defined cellular activity. Sci. Rep..

[30] Tam W.L., Freitas M.L., Chen X., Lesage R., Van Hoven I., Leysen E., Kerckhofs G., Bosmans K., Chai Y.C., Yamashita A., Tsumaki N., Geris L., Roberts S.J., Luyten F.P. (2021). Human pluripotent stem cell-derived cartilaginous organoids promote scaffold-free healing of critical size long bone defects. Stem Cell Res. Ther..

[31] Chen S., Chen X., Geng Z., Su J. (2022). The horizon of bone organoid: a perspective on construction and application. Bioact. Mater..

[32] Evrard R., Manon J., Maistriaux L., Rafferty C., Fieve L., Heller U., Cornu O., Gianello P., Schubert T., Lengele B. (2024). Decellularization of massive bone allografts by perfusion: a new protocol for tissue engineering. Tissue Eng. Part A.

[33] Zhong S., Lan Y., Liu J., Seng T.M., Hou Z., Zheng Q., Fu S., Bao D. (2025). Advances focusing on the application of decellularization methods in tendon-bone healing. J. Adv. Res..

[34] Al Q.A., Rani K., Syarif J., AlKawas S., Sheikh A.H.S., Samsudin A.R., Azlina A. (2023). Evaluation of decellularization process for developing osteogenic bovine cancellous bone scaffolds in-vitro. PLoS One.

[35] Dantas L.R., Ribeiro V., Kraft L., Pinho R.A., Suss P.H., Vasconcellos F., de Noronha L., Tuon F.F. (2022). Collagen matrices are preserved following decellularization of a bovine bone scaffold. Cell Tissue Bank..

[36] Perez M.L., Castells-Sala C., Lopez-Chicon P., Nieto-Nicolau N., Aiti A., Farinas O., Casaroli-Marano R.P., Porta O., Vilarrodona A. (2021). Fast protocol for the processing of split-thickness skin into decellularized human dermal matrix. Tissue Cell.

[37] Kirsner R.S., Bohn G., Driver V.R., Mills J.S., Nanney L.B., Williams M.L., Wu S.C. (2015). Human acellular dermal wound matrix: evidence and experience. Int. Wound J..

[38] Salhotra A., Shah H.N., Levi B., Longaker M.T. (2020). Mechanisms of bone development and repair. Nat. Rev. Mol. Cell Biol..

[39] Emami A., Arabpour Z., Izadi E. (2025). Extracellular vesicles: essential agents in critical bone defect repair and therapeutic enhancement. Mol. Biol. Rep..

[40] Vasileva R., Chaprazov T. (2023). Bone healing of critical-sized femoral defects in rats treated with erythropoietin alone or in combination with xenograft. Vet. Sci..

[41] Baldwin P., Li D.J., Auston D.A., Mir H.S., Yoon R.S., Koval K.J. (2019). Autograft, allograft, and bone graft substitutes: clinical evidence and indications for use in the setting of orthopaedic trauma surgery. J. Orthop. Trauma.

[42] Khanbazi M.H., Bigham-Sadegh A., Oryan A., Meimandi-Parizi A., Jannesar A.M. (2024). The effects of allogeneic and xenogeneic lyophilized leukocyte-and platelet-rich fibrin on bone healing in rat. Injury.

[43] Wubneh A., Tsekoura E.K., Ayranci C., Uludag H. (2018). Current state of fabrication technologies and materials for bone tissue engineering. Acta Biomater..

[44] Kim W., Jang C.H., Kim G. (2022). Bone tissue engineering supported by bioprinted cell constructs with endothelial cell spheroids. Theranostics.

[45] Jasim S.A., Bokov D.O., Suksatan W., Alsaikhan F., Jawad M.A., Sharma S.K., Chupradit S., Thangavelu L. (2023). Organoid models of heart diseases: find a new channel in improvements of cardiac regenerative medicine. Curr. Med. Chem..

[46] Nikolaev M., Mitrofanova O., Broguiere N., Geraldo S., Dutta D., Tabata Y., Elci B., Brandenberg N., Kolotuev I., Gjorevski N., Clevers H., Lutolf M.P. (2020). Homeostatic mini-intestines through scaffold-guided organoid morphogenesis. Nature.

[47] Liu H., Su J. (2023). Organoid extracellular vesicle-based therapeutic strategies for bone therapy. Biomater. Transl..

[48] Frenz-Wiessner S., Fairley S.D., Buser M., Goek I., Salewskij K., Jonsson G., Illig D., Zu P.B., Petersheim D., Li Y., Chen P.H., Kalauz M., Conca R., Sterr M., Geuder J., Mizoguchi Y., Megens R., Linder M.I., Kotlarz D., Rudelius M., Penninger J.M., Marr C., Klein C. (2024). Generation of complex bone marrow organoids from human induced pluripotent stem cells. Nat. Methods.

[49] Khan A.O., Rodriguez-Romera A., Reyat J.S., Olijnik A.A., Colombo M., Wang G., Wen W.X., Sousos N., Murphy L.C., Grygielska B., Perrella G., Mahony C.B., Ling R.E., Elliott N.E., Karali C.S., Stone A.P., Kemble S., Cutler E.A., Fielding A.K., Croft A.P., Bassett D., Poologasundarampillai G., Roy A., Gooding S., Rayes J., Machlus K.R., Psaila B. (2023). Human bone marrow organoids for disease modeling, discovery, and validation of therapeutic targets in hematologic malignancies. Cancer Discov..

[50] Xie C., Liang R., Ye J., Peng Z., Sun H., Zhu Q., Shen X., Hong Y., Wu H., Sun W., Yao X., Li J., Zhang S., Zhang X., Ouyang H. (2022). High-efficient engineering of osteo-callus organoids for rapid bone regeneration within one month. Biomaterials.

[51] Park Y., Cheong E., Kwak J.G., Carpenter R., Shim J.H., Lee J. (2021). Trabecular bone organoid model for studying the regulation of localized bone remodeling. Sci. Adv..

[52] Bargavi P., Ramya R., Chitra S., Vijayakumari S., Riju C.R., Durgalakshmi D., Rajashree P., Balakumar S. (2020). Bioactive, degradable and multi-functional three-dimensional membranous scaffolds of bioglass and alginate composites for tissue regenerative applications. Biomater. Sci..

[53] Gai T., Zhang H., Hu Y., Li R., Wang J., Chen X., Wang J., Chen Z., Jing Y., Wang C., Bai L., Wang X., Su J. (2025). Sequential construction of vascularized and mineralized bone organoids using engineered ECM-DNA-CPO-based bionic matrix for efficient bone regeneration. Bioact. Mater..

[54] Vallmajo-Martin Q., Kivelio A.S., Metzger S., Milleret V., Lienemann P.S., Carrara B.M., Millan C., Ghayor C., Ochsenbein-Koelble N., Ehrbar M. (2025). Undifferentiated human amniotic fluid progenitor cells promote bone regeneration in vivo. Adv. Healthcare Mater..

[55] Shafiee A., Sun J., Ahmed I.A., Phua F., Rossi G.R., Lin C.Y., Souza-Fonseca-Guimaraes F., Wolvetang E.J., Brown J., Khosrotehrani K. (2024). Development of physiologically relevant skin organoids from human induced pluripotent stem cells. Small.

[56] Jiang Y., Li Y., Duan L., Jiang B. (2025). Amniotic fluid-derived stem cells: an overlooked source of stem cells for translational research. DNA Cell Biol..

[57] Chen R., Dong H., Raval D., Maridas D., Baroi S., Chen K., Hu D., Berry S.R., Baron R., Greenblatt M.B., Gori F. (2023). Sfrp4 is required to maintain ctsk-lineage periosteal stem cell niche function. Proc. Natl. Acad. Sci. U. S. A.

[58] Hofbauer P., Jahnel S.M., Papai N., Giesshammer M., Deyett A., Schmidt C., Penc M., Tavernini K., Grdseloff N., Meledeth C., Ginistrelli L.C., Ctortecka C., Salic S., Novatchkova M., Mendjan S. (2021). Cardioids reveal self-organizing principles of human cardiogenesis. Cell.

[59] Qian S., Mao J., Liu Z., Zhao B., Zhao Q., Lu B., Zhang L., Mao X., Cheng L., Cui W., Zhang Y., Sun X. (2022). Stem cells for organoids. Smart Med..

[60] Munera J.O., Kechele D.O., Bouffi C., Qu N., Jing R., Maity P., Enriquez J.R., Han L., Campbell I., Mahe M.M., McCauley H.A., Zhang X., Sundaram N., Hudson J.R., Zarsozo-Lacoste A., Pradhan S., Tominaga K., Sanchez J.G., Weiss A.A., Chatuvedi P., Spence J.R., Hachimi M., North T., Daley G.Q., Mayhew C.N., Hu Y.C., Takebe T., Helmrath M.A., Wells J.M. (2023). Development of functional resident macrophages in human pluripotent stem cell-derived colonic organoids and human fetal colon. Cell Stem Cell.

[61] Xing F., Li L., Sun J., Liu G., Duan X., Chen J., Liu M., Long Y., Xiang Z. (2019). Surface mineralized biphasic calcium phosphate ceramics loaded with urine-derived stem cells are effective in bone regeneration. J. Orthop. Surg. Res..

[62] Wu S., Chen Z., Yu X., Duan X., Chen J., Liu G., Gong M., Xing F., Sun J., Huang S., Xiang Z. (2022). A sustained release of BMP2 in urine-derived stem cells enhances the osteogenic differentiation and the potential of bone regeneration. Regen. Biomater..

[63] Zhang C., Jing Y., Wang J., Xia Z., Lai Y., Bai L., Su J. (2024). Skeletal organoids. Biomater Transl.

[64] Faeed M., Ghiasvand M., Fareghzadeh B., Taghiyar L. (2024). Osteochondral organoids: current advances, applications, and upcoming challenges. Stem Cell Res. Ther..

[65] Qiao F., Zou Y., Bie B., Lv Y. (2024). Dual siRNA-Loaded cell membrane functionalized matrix facilitates bone regeneration with angiogenesis and neurogenesis. Small.

[66] Polak M., Karbowniczek J.E., Stachewicz U. (2024). Strategies in electrospun polymer and hybrid scaffolds for enhanced cell integration and vascularization for bone tissue engineering and organoids. Wiley Interdiscip. Rev. Nanomed. Nanobiotechnol..

