# Supplementary material for: Urine-derived stem cells efficiently assemble into micro-bone organoids supported by decellularized bone matrix microparticles for rapidly repairing bone defects through direct filling and paracrine functions
Source: Mater Today Bio. 2025 Nov 7;35:102533. doi: 10.1016/j.mtbio.2025.102533 (PMC12639860; doi:10.1016/j.mtbio.2025.102533)
Supplement: Multimedia component 1 [file mmc1.docx]

**Supporting Information**


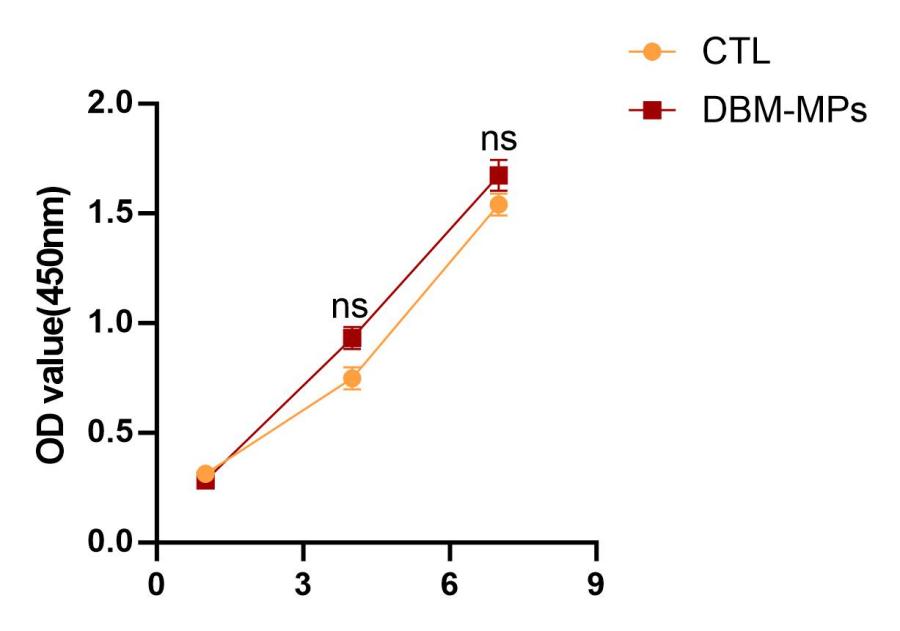


**Figure S1.** CCK-8 assay was performed to evaluate cell viability when DBM-MPs were co-cultured with BSMCs for 1, 4, and 7 days.


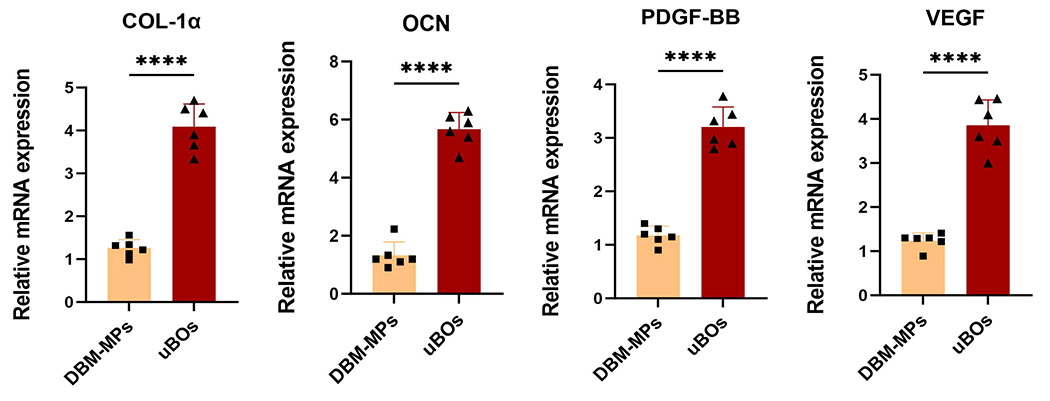


**Figure S2.** qRT-PCR was used to detect the expression levels of osteogenic markers (*COL-1α*, *OCN*) and angiogenic markers (*PDGF-BB*, *VEGF*) in the subcutaneous tissues of nude mice.

**Table S1**. Primer sequencesx

| Primer name | Primer sequence (5' to 3') | Species |
| --- | --- | --- |
| H-*RUNX2* Forward | GACCAGTCTTACCCCTCCTA | Human |
| H-*RUNX2* Reverse | GGCAGTGTCATCATCTGAAA | Human |
| H-*HIF-1α* Forward | GGACAGCCTCACCAAACAGA | Human |
| H-*HIF-1α* Reverse | TAGCTGCATGATCGTCTGGC | Human |
| H-*OCN* Forward | CTCTCTCTGCTCACTCTGCT | Human |
| H-*OCN* Reverse | GACTGAGGCTCCAAGGTAG | Human |
| H-*ALP* Forward | GCTTTAAACCCAGACACAAG | Human |
| H-*ALP* Reverse | AAGAAGAAGCCTTTGAGGTT | Human |
| H-*COL-1α* Forward | GAGAGCATGACCGATGGATT | Human |
| H-*COL-1α* Reverse | CCTTCTTGAGGTTGCCAGTC | Human |
| H-*VEGF* Forward | GTAACGATGAAG CCCTGGAGT | Human |
| H-*VEGF* Reverse | TGTTCTGTCTTTCTT TGGTCTGC | Human |
| H-*GAPDH* Forward | GAAGGTCGGAGTCAACGGATTT | Human |
| H-*GAPDH* Reverse | CTGGAAGATGGTGATGGGATTTC | Human |
| M-*OCN* Forward | AGCCTTTGTGTCCAAGCA | Mouse |
| M-*OCN* Reverse | CCAGCCATTGATACAGGTAG | Mouse |
| M-*PDGF-BB* Forward | ATATTCCAACCCAGCCACGA | Mouse |
| M-*PDGF-BB* Reverse | TGAACAGTGCTCCCTGCATA | Mouse |
| M-*VEGF* Forward | CCCGACAGGGAAGACAAT | Mouse |
| M-*VEGF* Reverse | TCTGGAAGTGAGCCAACG | Mouse |
| M-*COL-1α* Forward | GACATCCCACCAATCACCTG | Mouse |
| M-*COL-1α* Reverse | CGTCATCGCACAACACCTT | Mouse |
| M-*GAPDH* Forward | GGCAAATTCAACGGCACAGTCAAG | Mouse |
| M-*GAPDH* Reverse | TCGCTCCTGGAAGATGGTGATGG | Mouse |
| R-*RUNX2* Forward | TCGTCAGCGTCCTATCAGTTCC | Rat |
| R-*RUNX2* Reverse | CTTCCATCAGCGTCAACACCATC | Rat |
| R-*OCN* Forward | CCTCACACTCCTCGCCCTATT | Rat |
| R-*OCN* Reverse | CCCTCCTGCTTGGACACAAA | Rat |
| R-*PDGF-BB* Forward | TTCCTGCCTCTCTGCTGCTAC | Rat |
| R-*PDGF-BB* Reverse | ATCTTCGTCTACGGAGTCTCTGTG | Rat |
| R-*VEGF* Forward | GAGCGGAGAAAGCATTTGTTTGTC | Rat |
| R-*VEGF* Reverse | CGCCTTGGCTTGTCACATCTG | Rat |
| R- *GAPDH* Forward | GTCCATGCCATCACTGCCACTC | Rat |
| R- *GAPDH* Reverse | CGCCTGCTTCACCACCTTCTTG | Rat |
